# Regulation of dhurrin pathway gene expression during *Sorghum bicolor* development

**DOI:** 10.1007/s00425-021-03774-2

**Published:** 2021-11-11

**Authors:** Roslyn M. Gleadow, Brian A. McKinley, Cecilia K. Blomstedt, Austin C. Lamb, Birger Lindberg Møller, John E. Mullet

**Affiliations:** 1 School of Biological Sciences, Monash University, Clayton, VIC Australia; 2 Department of Plant Biochemistry and Biophysics, Texas A&M University, College Station, TX USA; 3 Plant Biochemistry Laboratory, Department of Plant and Environmental Sciences, University of Copenhagen, Copenhagen, Denmark

**Keywords:** Cyanogenic glucosides, Diel cycle, Hydrogen cyanide, Ontogeny, Plant defence, RNA-seq, *Sorghum bicolor*

## Abstract

**Main conclusion:**

**Developmental and organ-specific expression of genes in dhurrin biosynthesis, bio-activation, and recycling offers dynamic metabolic responses optimizing growth and defence responses in Sorghum**.

**Abstract:**

Plant defence models evaluate the costs and benefits of resource investments at different stages in the life cycle. Poor understanding of the molecular regulation of defence deployment and remobilization hampers accuracy of the predictions. Cyanogenic glucosides, such as dhurrin are phytoanticipins that release hydrogen cyanide upon bio-activation. In this study, RNA-seq was used to investigate the expression of genes involved in the biosynthesis, bio-activation and recycling of dhurrin in *Sorghum bicolor*. Genes involved in dhurrin biosynthesis were highly expressed in all young developing vegetative tissues (leaves, leaf sheath, roots, stems), tiller buds and imbibing seeds and showed gene specific peaks of expression in leaves during diel cycles. Genes involved in dhurrin bio-activation were expressed early in organ development with organ-specific expression patterns. Genes involved in recycling were expressed at similar levels in the different organ during development, although post-floral initiation when nutrients are remobilized for grain filling, expression of *GSTL1* decreased > tenfold in leaves and *NITB2* increased > tenfold in stems. Results are consistent with the establishment of a pre-emptive defence in young tissues and regulated recycling related to organ senescence and increased demand for nitrogen during grain filling. This detailed characterization of the transcriptional regulation of dhurrin biosynthesis, bioactivation and remobilization genes during organ and plant development will aid elucidation of gene regulatory networks and signalling pathways that modulate gene expression and dhurrin levels. In-depth knowledge of dhurrin metabolism could improve the yield, nitrogen use efficiency and stress resilience of *Sorghum*.

**Supplementary Information:**

The online version contains supplementary material available at 10.1007/s00425-021-03774-2.

## Introduction

Ontogenetic trajectories in defence have been described in hundreds of species of plants. Typically, defence compound levels are high in young plants, with overall concentration decreasing as plants mature (e.g. Gleadow and Woodrow 2002). Such changes may be adaptive, providing protection for the meristems and newly formed organs. When evaluating possible trade-offs between costs and benefits of investment in biotic defences, there are a number of other considerations that could moderate and/or off-set the cost of production (Neilson et al. 2013; Gleadow and Møller 2014; Blomstedt et al. 2018): (1) Are there developmental stage dependent metabolic constraints? (2) Are light or mineral nutrients limiting? (3) How effective are the defence compounds produced in deterring herbivores? and (4) Could these compounds have other potentially beneficial functions? Allocation models of investment provide mechanistic explanations of why costs of defence change as plants mature (Boege and Marquis 2005) and under different environmental conditions (Gleadow et al. 1998) but do not address the underlying molecular regulatory mechanisms.

Cyanogenic glucosides are present in over 2500 plant species and are known to be effective in herbivore defence (Tattersall et al. 2001a; Gleadow and Woodrow 2002; Ballhorn et al. 2006). Cyanogenic glucosides are classified as phytoanticipins, i.e. the defensive agent is released when required from a constitutive progenitor. In this case, a hydrogen cyanide ‘bomb’ is detonated when the cells in which they are stored are disrupted, e.g. by mastication, mixing the components with specific hydrolyzing enzymes (Gleadow and Møller 2014). As with many chemical defence systems, the costs of deployment in terms of a growth sacrifice are small, and usually only detectable under resource-limiting conditions, if at all (Blomstedt et al. 2018; Simon et al. 2010; Sohail et al. 2020; Gershenzon 1989). This lack of apparent cost could be because the costs of production are tiny (Gershenzon 1989) or off-set by other metabolic or physiological benefits (Neilson et al. 2013).

Dhurrin, the cyanogenic glucoside present in *Sorghum bicolor*, has been widely studied and established as a model for testing defence theories (Miller et al. 2014; Blomstedt et al. 2018; Gleadow et al. 2016). While the biosynthetic pathway is well characterised (Halkier and Møller 1989; Kahn et al. 1997; Møller and Conn 1979), little is known about the transcriptional regulation of genes in the pathway (Busk and Møller 2002). The concentration of dhurrin in sorghum is dependent on the genotype (Emendack et al. 2018; Hayes et al. 2015; Burke et al. 2013), tissue type (O’Donnell et al. 2013), plant age (Blomstedt et al. 2018), stage of development (Miller et al. 2014) and growing conditions (O’Donnell et al. 2013; Gleadow et al. 2016; Rosati et al. 2019). Information on pathway gene expression in organs, tissues and cells during development and under different environmental conditions is needed to provide information for gene regulatory network analysis and to elucidate the signalling pathways that regulate expression of the genes involved in dhurrin synthesis, transport, hydrolysis and re-mobilization.

**Fig. 1:**
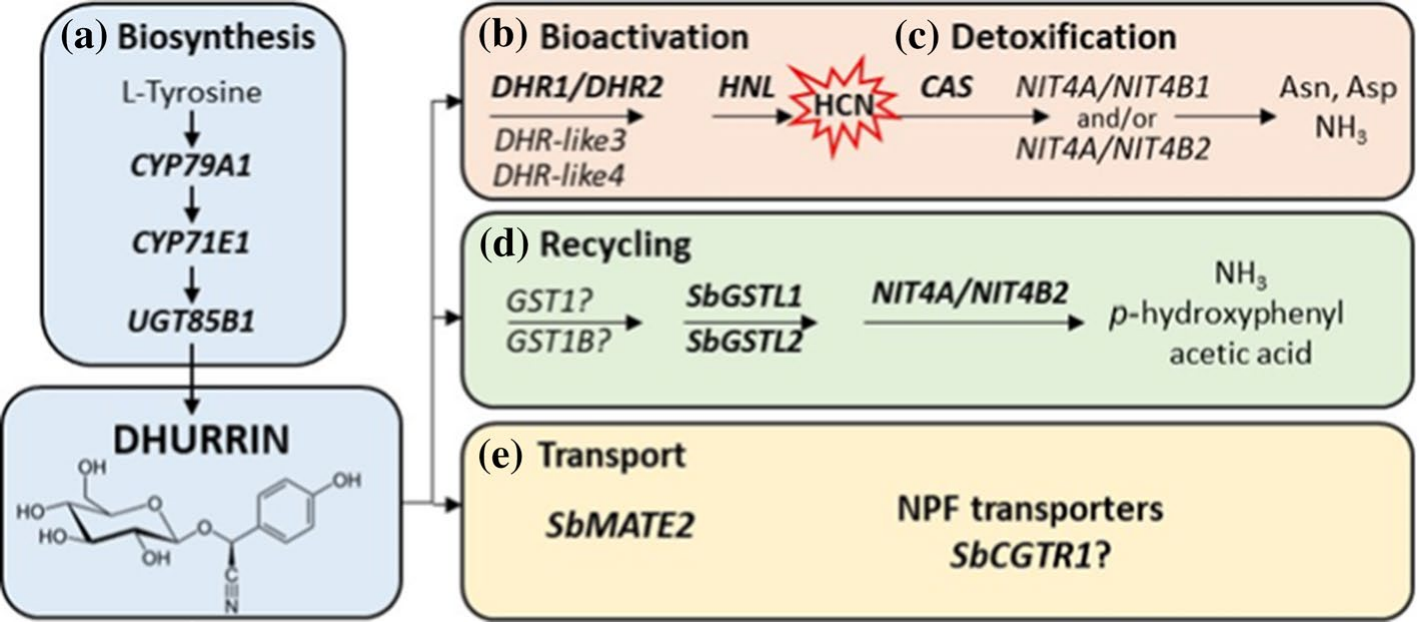
Overview of the genes involved in dhurrin biosynthesis, recycling, transport, bioactivation and in prevention of auto-toxicity. **a** Dhurrin is synthesised from tyrosine by the sequential action of three key enzymes. **b** Bioactivation of dhurrin to release a hydrogen cyanide bomb occurs upon tissue disruption by the action of specific β-glucosidases (DHR) and an α-hydroxynitrile lyase (HNL). **c** Auto-toxicity is prevented by β-cyanoalanine synthase and nitrilases retrieving the nitrogen of the hydrogen cyanide for production of amino acids and ammonia. **d** Endogenous recycling pathway mediated by glutathione transferases and a heteromeric NIT4A/NIT4B2 nitrilase resulting in formation of ammonia and *p-*hydroxyphenylacetic acid without the release of HCN. **e** Dhurrin synthesis occurs in the cytosol and two potential genes, *SbMATE2* and *SbCGTR1*, may be involved in dhurrin transport to sites of storage within the cell or to other parts of the plant. Abbreviations: *Asn* asparagine; *Asp* aspartic acid; *DHR* dhurrinase; *NH*_*3*_, ammonia

Dhurrin ((*S*)-*p*-hydroxymandelonitrile-β-D-glucopyranoside) is biosynthesized from tyrosine in a process catalysed by the sequential action of two cytochrome P450s (CYP79A1 and CYP71E1) and a UDP-glucosyltransferase (UGT85B1) with the NADPH-dependent cytochrome P450 oxidoreductase (POR) serving as an electron donor to the P450s (Fig. 1) (Kahn et al. 1997; Bak et al. 1998; Møller and Conn 1979; Jones et al. 1999). These enzymes form a metabolon which facilitates channelled conversion of the intermediates into dhurrin formation and prevents release of toxic intermediates and undesired metabolic cross-talk (Jørgensen et al. 2005; Kristensen et al. 2005; Nielsen et al. 2008; Laursen et al. 2015). The genes encoding the enzymes in this metabolon (except *POR*) are clustered on chromosome 1 together with *SbMATE2* and a glutathione S-transferase (*GST*) (Darbani et al. 2016; Hayes et al. 2016; Takos et al. 2011). Genes involved in dhurrin biosynthesis located in the cluster are co-expressed and protein production is regulated in part at the transcriptional level (Takos and Rook 2012; Darbani et al. 2016; Busk and Møller 2002). In *S. bicolor*, dhurrin synthesis is highly active in young leaf and stem tissues during vegetative plant development (Busk and Møller 2002).

Dhurrin may also be recycled in a glutathione transferase-catalysed process proceeding without concomitant release of HCN (Pičmanová et al. 2015; Bjarnholt et al. 2018; Jenrich et al. 2007). In the recycling process, the glucose moiety of dhurrin is replaced by glutathione, either spontaneously or potentially by a glutathione transferase (e.g. GST1 within the biosynthesis cluster). The glutathione conjugate is subsequently cleaved by GSTs of the lambda class (GSTL1/2) resulting in the production of *p-*hydroxyphenylacetonitrile, which through the action of the NIT4A/NIT4B2 heterodimer is converted into *p-*hydroxyphenylacetic acid and ammonium. The possibility of recycling without the release of toxic intermediates increases the likelihood that dhurrin could have other roles, depending on stage of plant development and environmental conditions (Gleadow and Møller 2014; Bjarnholt et al. 2018).

The sub-cellular storage site of dhurrin has previously been reported to be the vacuole (Saunders and Conn 1978) but more recent data points to the cytosol as the storage site, maybe in the form of a biocondensate (Møller and Laursen 2021; Heraud et al. 2018; Knudsen et al. 2018, 2020). Proper storage of dhurrin may involve transporters. In this context, dhurrin may be transported to the vacuole or other vesicle enclosed compartments by *Sb*MATE2, a transporter of the multidrug and toxic compound extrusion (MATE) family (Darbani et al. 2016). In addition, a member of the nitrate and peptide family (NPF) of transporters has been identified in cassava, another well-studied cyanogenic plant, to exhibit high substrate specificity for linamarin, the main cyanogenic glucoside in that species [MeCGTR1; (Jørgensen et al. 2017)].

Upon cell destruction and exerting its function as part of a two-component defence system, dhurrin is hydrolysed and the hydrogen cyanide is rapidly released with co-production of stoichiometric amounts of *p-*hydroxybenzaldehyde. This process is catalysed by specific β—glucosidases (dhurrinases) and α-hydroxynitrilases (HNLs). Two dhurrinases (DHR1 and DHR2) have been identified that exhibit tissue-specific expression (Hösel et al. 1987; Cicek and Esen 1998). Sequence analysis has identified two additional dhurrinase-like genes and all four DHR genes are co-located (within ~ 630 kb) on chromosome 8 (Hayes et al. 2015, 2016; Krothapalli et al. 2013). The prevalence of two or more differentially expressed β-glucosidase isoenzymes in most plants suggests that the two-component defence system exemplified by cyanogenic glucosides is spatially and/or temporally regulated (Morant et al. 2008b). The α-hydroxynitrile intermediate formed is converted to hydrogen cyanide and *p-*hydroxybenzaldehyde via the action of α-hydroxynitrile lyase (HNL), although at pH > 6, the dissociation of the α-hydroxynitrile intermediate also proceeds non-enzymatically (Poulton 1990). To avoid auto-toxicity, the released hydrogen cyanide can be detoxified and reincorporated into primary metabolism in a process mediated by β-cyanoalanine synthase (CAS) and the nitrilases NIT4A, NIT4B1 or NIT4B2 (Fig. 1).

*Sorghum bicolor* (L.) Moench is one of the world’s leading cereal crops and a valuable forage crop, particularly in the semi-arid tropics. Photoperiod-sensitive sorghum is also a good source of biomass for bioenergy production due to the high productivity of this C4 grass and the crop’s drought tolerance, allowing production on marginal annual cropland. Bioenergy sorghum hybrids have been developed and under field conditions have shown good greenhouse gas mitigation potential (Olson et al. 2012; Rooney et al. 2007; Gill et al. 2014; Mullet et al. 2014; McKinley et al. 2018b). Improvements in biotic stress resilience and nitrogen use efficiency would benefit all types of sorghum crops.

The availability of sequenced and annotated sorghum genomes (Paterson et al. 2009; Mace et al. 2013; McKinley et al. 2018a; Cooper et al. 2019) presents new opportunities for improving *S. bicolor* for grain, forage and bioenergy production (McCormick et al. 2018; Kebrom et al. 2017, 2020; McKinley et al. 2018a, 2018b). In this study, transcriptome analysis aided by RNA-seq was used to compare the expression of the key genes involved in sorghum dhurrin biosynthesis, bio-activation, recycling, and transport in the juvenile and adult-phase vegetative tissues and from floral initiation until anthesis. Diel expression of genes in the dhurrin pathway was characterized over two light and one dark cycle in leaves of adult-phase vegetative plants.

## Materials and methods

### Plant material, growing conditions and sampling for RNA-seq study

**Fig. 2:**
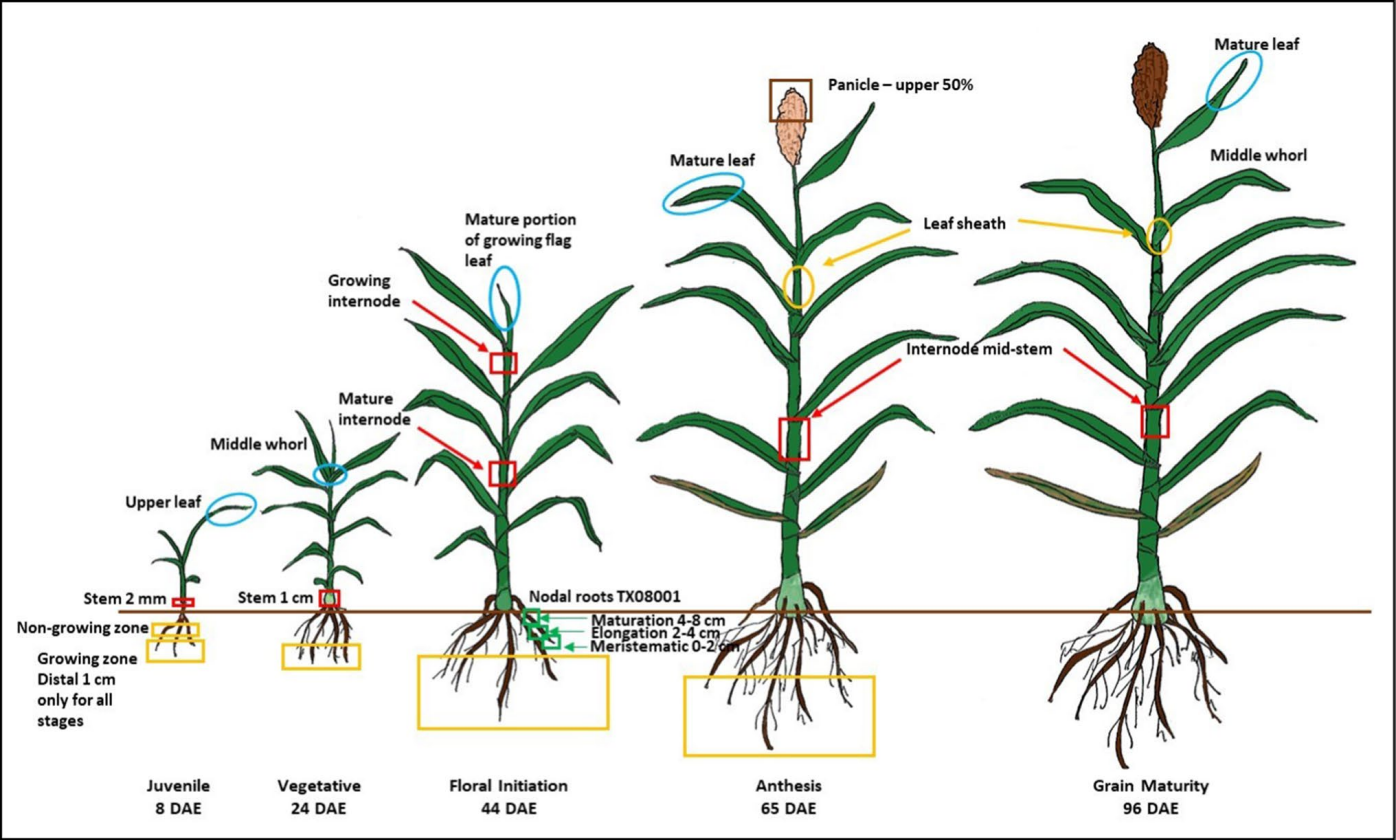
Schematic showing the developmental stages of *Sorghum bicolor* BTx623 and the tissues selected for RNA-seq analysis. The nodal root tissue is shown on this figure, but the tissue was taken from the high bioenergy cultivar TX08001. DAE—days after emergence. The colour of the square or oval denoting the tissue samples indicates the different tissue selected and corresponds to the expression data in tables; Green—nodal roots Table 2a; Yellow square—root Table 2b; Red—stem Table 3a; Yellow circle—Table 4; Blue—leaves; Table 5

RNA-seq data were derived primarily from *S. bicolor* BTx623, an important public breeding line and the genotype used for the first sorghum reference genome sequence (McCormick et al. 2018; Paterson et al. 2009). Tissue and development-dependent analyses of RNA-seq profiles related to dhurrin biosynthesis, bio-activation and detoxification, recycling and transport were carried out using the methodologies described in McCormick et al. (2018). For the diel experiment, BTx623 plants were grown in a growth chamber (300 μmol, 30 °C day/23 °C night, 50% relative humidity) and entrained to a 14 h photoperiod for 39 day (adult v-phase) (Murphy et al. 2011). Fully expanded adult leaf blades were harvested every 3 h, with the first time-point (0) within 10 min after lights were turned on in the morning and continuing every 3 h for 36 h. RNA-seq data were likewise derived from the photoperiod-sensitive bioenergy sorghum genotype R.07020 (Kebrom et al. 2017; Casto et al. 2018) and from the bioenergy sorghum hybrid TX08001 (Kebrom and Mullet 2016). RNA-seq data were generated from roots of the bioenergy hybrid TX08001 grown to 120 DAE in a Westwood silt loam soil under field conditions (see Fig. 2 for tissue locations) and from the tiller buds of leaves of the 100 M cultivar grown in growth chambers, as described above. 100 M is a photoperiod-sensitive line (*Ma1Ma2Ma3Ma4Ma5ma6*) that is part of a panel of genotypes that vary in flowering time (Quinby 1974). Sorghum genotypes that are photoperiod-sensitive (i.e., 100 M, R07020, TX08001) exhibit delayed flowering when grown in long days (> 12.4 h day lengths), whereas photoperiod-insensitive genotypes such as BTx623 flower in about 65–70 days in both long and short days. The genotypes selected for this study represent breeding lines or hybrids used in production of grain sorghum (i.e., BTx623) and bioenergy sorghum (i.e., TX08001, R07020) that have been the subject of prior transcriptome analysis. Seed for all genotypes was obtained from the Sorghum Breeding Lab at Texas A&M University in College Station, TX.

### RNA extraction, template preparation and sequencing

The RNA-seq data reported on in this study were generated from multiple experiments conducted over several years focused on characterizing organ, tissue and developmental patterns of gene expression in sorghum (Kebrom et al. 2017, 2020; McKinley et al. 2016, 2018a; McCormick et al. 2018; Casto et al. 2018). Following tissue harvest, three biological replicates of each tissue were stored at − 80 °C until RNA extraction. Tissues were then pulverized by mortar and pestle and total RNA was extracted from ~ 100 mg of frozen tissue solubilized with 1 mL of Trizol reagent. RNA was isolated and purified using the Direct-zol RNA Miniprep kit (Zymo Research, Irvine CA). Samples were then aliquoted into 96-well plates and shipped to the Joint Genome Institute for sequencing using an automated sequencing pipeline to a depth of 30–50 M reads per sample (McCormick et al. 2018).

### RNA-seq analysis

For all RNA-seq experiments, reads were aligned to the *S. bicolor* V3.1 genome using the HISAT2 aligner (Daehwan et al. 2015; McCormick et al. 2018). Transcriptome assembly and Transcripts Per Million Reads (TPM) normalization were performed using StringTie version 1.3 (Pertea et al. 2015). Analysis and visualization of gene expression data were conducted using TPM normalized data. The prepDE.py script was used to convert nucleotide coverage data from StringTie into reads that are compatible with differential expression statistical packages that use conventional raw reads. Differential expression analysis (DE) was calculated using the edgeR package to determine the fold change (FC) between samples, and the false discovery rate (FDR)—adjusted *p* values. The fold change displayed in the tables is the absolute value of the fold change between the maximum and minimum expression values. The FDR-adjusted p value also corresponds to the maximum and minimum expression values for each gene analysed. In some cases, zero expression at one time point when compared to expression at another time-point led to a very high differential in expression values. To minimize this effect, in instances with very high DE values, samples with < 1 TPM were set to a TPM = 1 and the fold change was recalculated as FC = maxTPM/1 (McKinley et al. 2016). These instances are indicated with the “ > ” sign in front of the fold change values since the actual fold change is assumed to be some value greater than the value displayed. Functional analysis of gene families utilized protein family annotations (pfam) as well as gene functional annotations generated by DOE-JGI for the *S. bicolor* V3.1 genome available through Phytozome (https://phytozome.jgi.doe.gov/pz/portal.html#!info?alias=Org_Sbicolor) (McCormick et al. 2018).

### Basis for selection of genes for analysis

Transcript levels of genes with an identified role in dhurrin biosynthesis, hydrogen cyanide bio-activation and detoxification, recycling and transport (Fig. 1) were measured (Gleadow and Møller 2014; Darbani et al. 2016; Hayes et al. 2015; Nielsen et al. 2016; Akbudak et al. 2019; Bjarnholt et al. 2018). Additional details are available in Supplementary Information (Methods S1).

### Phylogenetic analysis of dhurrin pathway genes

The cytochrome P450s, CYP79A1 and CYP71E1 have been well characterised (Fig. 1) (Gleadow and Møller 2014; Bak et al. 2000; Tattersall et al. 2001b; Thorsøe et al. 2005; Kahn et al. 1999). In this study, phylogenetic analysis was done to check or expand the cytochrome P450s (PF0067) and the glycoside hydrolases (PF00232). Genes related to either *CYP79A1* or *DHR1* were initially identified by selecting genes that encode proteins that possess the respective pfam domains present in the relevant gene. The protein sequences encoded by these genes were obtained from Biomart, hosted by the Phytozome database (https://phytozome.jgi.doe.gov/biomart). Multiple sequence alignment and generation of phylogenetic trees using the neighbor-joining method were performed using Clustal Omega using default settings (Madeira et al. 2019). Phylogenetic trees were further rendered using the ggtree and Treeio R packages (Yu et al. 2017). Additional putative DHR encoding genes were identified based on shared membership within a clade containing the query genes of interest. Sequence alignment of DHR1 (Sobic.008G079800.1) with other members of the glycoside hydrolases identified DHR2 (Sobic.008G08400.1), DHR-like3 (Sobic.008G08100) and DHR-like4 (Sobic.008G080600) as the most closely related genes in the sorghum genome with alignment occurring across the entire length of the DHR1 sequence and very low E-values (set to 0 in Phytozome) (Supplementary Fig. S1). The genes in clades adjacent to the DHR-clade aligned with higher E-values and with low sequence alignment over the N-terminal 75 amino acids of DHR1. Phylogenetic trees are given in supplementary Figure S2 and S3. GenBank accession numbers are listed in Table S7.

### Plant material and growing conditions for dhurrin analysis

Seeds of *S. bicolor* BTx623 were germinated and grown in pots under controlled greenhouse conditions at Monash University, Melbourne, Australia (coordinates: 37°54′36″S 145°08′02″E) from December 2019–February 2020 with day/night temperatures of 28 ± 2 °C and 18 ± 2 °C, respectively, and an average photoperiod of 14 h (average photosynthetic photon flux density of 430 ± 80 μmol quanta m^–2^ s^–1^). Leaf and nodal root tissue were sampled for dhurrin quantification. The leaves selected were: (1) the last fully emerged and unfurled leaf, classified as older; and (2) a single younger, unfurled leaf positioned two leaves immediately above the last fully unfurled leaf. In plants that had bolted, this younger leaf was the leaf penultimate to the flag leaf. Nodal roots were divided into four sections at different distances from the root tip and their hydrogen cyanide potential analysed (Supplementary Information Fig. S4).

The concentration of the cyanogenic glucoside dhurrin was determined spectrophotometrically from the hydrogen cyanide potential (HCNp) using the method of Woodrow et al. (2002). The hydrogen cyanide potential (HCNp) is used as a proxy for the dhurrin content, with each mg of HCN being equivalent to 11.5 mg of dhurrin in the plant tissue. Free hydrogen cyanide released as a result of ethylene biosynthesis was assumed to be negligible. Leaf discs were sampled from two leaves (young and old) on four individual *S. bicolor* BTx623 plants: two plants 47-day post sowing and two plants 51-day post sowing that had undergone floral transition and stem bolting. Three leaf discs from each sampled leaf were placed in duplicate vials in 0.1 M citrate buffer (pH 5.0) with 300 µL of β-glucosidase (almond emulsin, G4511, Sigma). A duplicate set of analyses were performed without the addition of exogenous β-glucosidase. The HCN released was captured as NaCN in a 1 M NaOH solution and measured via a colorimetric assay (Woodrow et al. 2002). The leaf discs were subsequently dried and weighed to calculate HCNp mg g^−1^ dry mass. Mean tissue hydrogen cyanide potentials were compared using ANOVA in Microsoft Excel.

### Statistical analysis

**Fig. 3:**
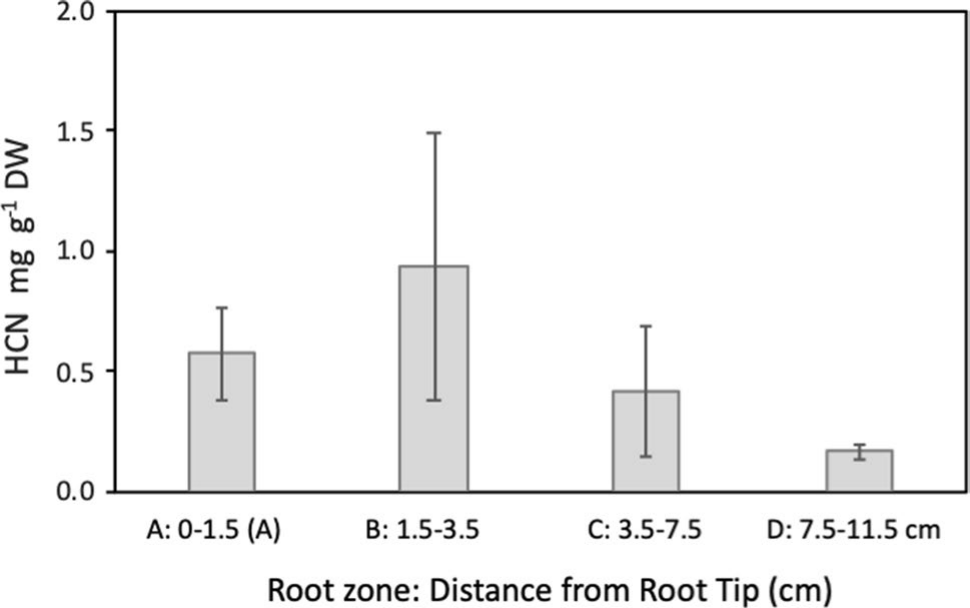
Hydrogen cyanide potential (HCNp) of nodal roots of *S. bicolor* BTx623 sampled at different distances from the tip, corresponding to the regions analysed by RNA-seq: section A—Meristematic region of root tip; Section B—Elongation zone; Section C—Mature zone 1; Section D—Mature zone 2, adjacent to stem. See supplementary figure (Fig. S4) for images of the roots used in this experiment

**Fig. 4:**
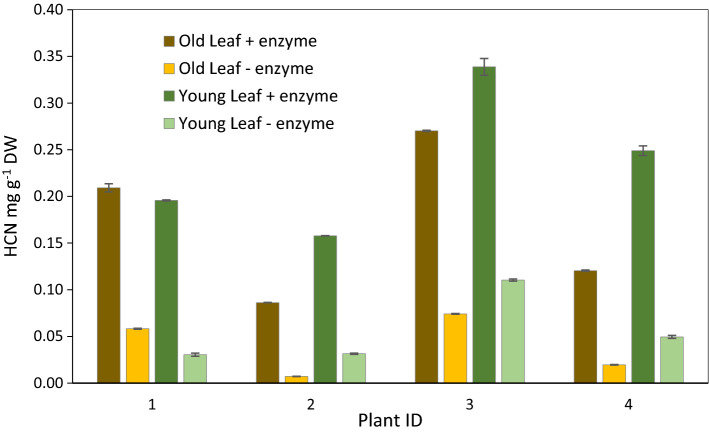
Variation in leaf HCNp among four *S. bicolor* BTx623 individuals (plant ID). Samples of fully expanded (old leaf) and young expanding leaves were tested with and without addition of exogenous almond emulsin containing β-glucosidases that are effective in catalysing the dhurrin hydrolysis

**Fig. 5:**
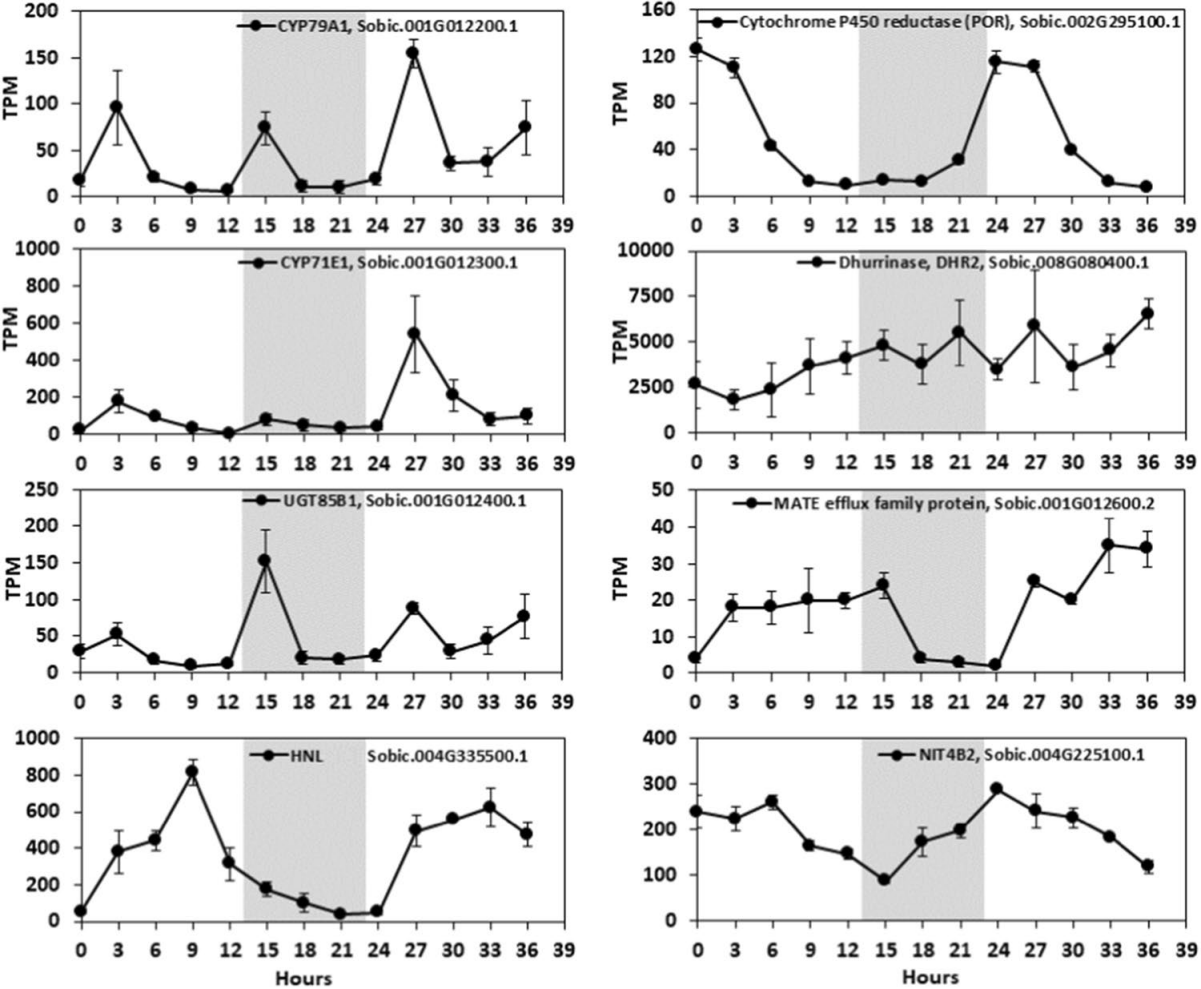
Diurnal expression of components of the dhurrin biosynthesis, bioactivation and recycling pathways and putative membrane transporter in leaves of BTx623 during vegetative growth. The day length during the experiment was 14 h/10 h, light/dark. White regions of the plots represent lights-on and grey-shaded regions represent lights-off during the experiment. Expression values are the means of three biological replicates (± 1 SEM). Data have been normalized as transcripts per million. The full data set for all genes in this is given Supplementary information (Table S6)

In all cases, RNA-seq sample data from each individual experiment shown are the mean of three biological replicates. The three biological replicates of juvenile BTx623 stems were composed of tissue from 5 plants per biological replicate to obtain enough tissue for RNA extraction. Similarly, the three biological replicates of the 100 M tiller bud samples were composed of six axillary buds per sample. For differential expression calculations, FDR-adjusted *p* values were calculated using the edgeR package. Error bars shown in Figs. 3, 4, and 5 are standard error of the mean (SEM). As stated previously, the significance between tissue hydrogen cyanide potentials was determined by calculating an F-statistic (ANOVA). Phylogenetic trees were constructed using the neighbor-joining method in Clustal Omega with default settings.

## Results

Data on the expression of genes involved in dhurrin biosynthesis (*CYP79A1, CYP71E1, UGT85B1* and *POR*), bioactivation (*DHR1*, *DHR2*, *DHR-like3*, *DHR-like4; HNL*), prevention of auto-toxicity (*CAS, NIT*) and endogenous recycling (*GST* lambda candidates and *NIT4A*/*NIT4B2*) during organ and plant development are presented. The nitrilases *NIT4A, NIT4B2* and *NIT4B1* are involved in detoxification as well as endogenous dhurrin recycling (Jenrich et al. 2007). We also analysed the expression of *SbMATE2*, which has been shown to transport dhurrin (Darbani et al. 2016) and a candidate nitrate/peptide family (NPF) transporter (*SbCGTR1*) that is the closest sorghum homolog of a transporter identified in cassava, *MeCGTR1*, specific for the cyanogenic glucoside, linamarin (Jørgensen et al. 2017). There were four key observations: (1) Expression of the genes involved in dhurrin biosynthesis, except *POR*, varied significantly during organ and plant development with highest expression in the active meristematic growth zones and juvenile tissues; (2) Expression of genes involved in dhurrin bio-activation was tissue dependent, with *DHR1* preferentially expressed in roots and stems, *DHR2* in leaf blades, *DHR-like3* in stem growing zones and *DHR-like4* in young leaf: leaf sheath collar tissue; (3) The nitrilases, *NIT4A* and *NIT4B2*, which form heterodimers specifically involved in the endogenous recycling of dhurrin, were expressed at higher levels in mature tissue of plants entering the reproductive stage; (4) Expression of dhurrin pathway genes varied during a diel cycle in leaves, with a peak of expression of the biosynthetic genes (*CYP79A1*, *CYP71E1*, *UGT85B1* including *POR*) occurring 3 h after onset of plant illumination at dawn and shortly after the onset of the dark period, whilst *SbMATE2* decreased at night and expression of *DHR2*, specific for bio-activation in the leaves, showed only small changes in expression amplitude during diel cycles. For clarity, data in tables have been limited to genes that showed significant variation in expression during organ or plant development or a diel cycle. Expanded tables that include genes with low or undetectable expression can be found in the Supplementary Information Tables S1–S6.

### Transcriptomic analysis of tissue from different organs and developmental stages

#### Dhurrin pathway gene expression increases during seed imbibition

Transcript levels of the dhurrin biosynthetic pathway genes *CYP79A1*, *CYP71E1* and *UGT85B1* were very low in dry seed, consistent with prior results (Nielsen et al. 2016). Transcript levels of *CYP79A1*, *CYP71E1* and *UGT85B1* increased in seeds imbibed for 24 h, especially the key gene *CYP79A1,* consistent with prior data showing rapid accumulation of dhurrin in emerging seedlings (Table 1) (Heraud et al. 2018; Halkier and Møller 1989; Busk and Møller 2002). Transcript levels of the *DHR1* increased with seed imbibition (Table 1). There was no change in the expression of hydroxynitrile lyase (*HNL*) in imbibed seed but the expression of *CAS C1* increased 15-fold (Table 1). The recycling genes *NIT4A/B2* both increased in expression during imbibition. A small increase in expression of *NIT4B1* involved in hydrogen cyanide detoxification is also observed (Supplementary information Table S1). The expression of the *SbMATE2* dhurrin transporter and the putative cyanogenic glucoside transporter, *SbCGTR1,* also increased following seed imbibition.

**Table 1:**
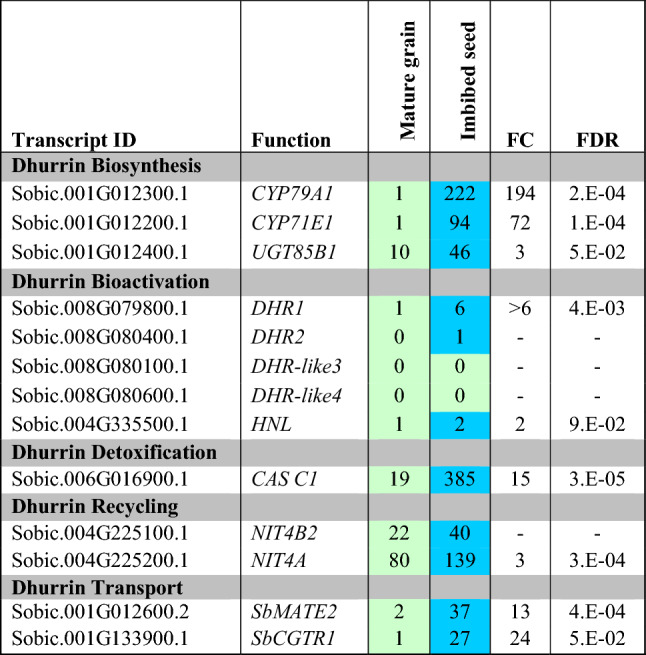
Heat map showing expression of transcripts for key cyanogenesis-related genes in mature grain (dry seeds) and imbibed seed of BTx623

*TPM* transcripts per million reads, *n* = 3, FC and FDR from edgeR. Genes with increased expression levels are shown in blue and reduced expression levels in green. Only data for genes with changes in expression are presented. Expanded tables that include genes with low or undetectable expression can be found in the Supplementary Information (Table S1)

#### Dhurrin pathway genes are expressed during tiller bud out growth

Tiller buds formed in the first leaf axil grow steadily in size for 6–8 day post germination prior to becoming dormant or entering into a phase of rapid leaf outgrowth (Kebrom and Mullet 2016). Dhurrin pathway genes involved in biosynthesis were highly expressed in tiller buds of the first leaf axil of 100 M at 8 day after germination (Table 7b; Supplementary information Table S1). *DHR2*, a gene differentially expressed in leaves, was expressed at very high levels in tiller buds, *DHR1* and *DHR-like3* at low levels, and *DHR-like4* expression was low or undetectable (Supplementary information Table S1). *HNL* and *CAS* are also expressed at high levels. In the recycling pathway, three GST genes are highly expressed including two transcripts derived from *GST1* (Table S1). However, these GSTs are likely to be involved in functions other than recycling as *GSTL1*, *GSTL2* and *NIT4A/4B2,* which have been identified in dhurrin recycling, show low or no expression (Table S1). Expression of the *SbMATE2* dhurrin transporter was also detected in tiller buds.

#### Dhurrin pathway gene expression during root and plant development

Nodal roots grow out from the stem and extend into the soil profile through tip growth. Expression of genes in the dhurrin pathway during nodal root development was analysed by collecting tissue from TX08001 nodal roots that had recently become embedded in the soil but prior to lateral root proliferation. Three regions of the developing nodal root were collected; ~ 2 cm of nodal root tissue that spans the root tip-growing zone, the adjacent ~ 2 cm of root tissue that may span a portion of the zone of elongation and 4 cm of more fully differentiated root tissue (Table 2a; Fig. 2 green boxes). Expression of *CYP79A1, CYP71E1* and *UGT85B1* was highest in root tissue that spans the root tip meristem/growing zone (Fig. 2a, Meristematic). Expression of these genes gradually decreased during maturation of nodal root tissues. *DHR1* was expressed at very high levels in the root tip-growing zone but at very low levels in older root tissue. *DHR-like 3* was expressed at low levels in the root tip-growing zone but not in more mature root tissues and expression of *DHR2* and *DHR-like4* was not detected in nodal roots. Expression of the genes involved in dhurrin recycling was 2–4-fold higher in root tissue just above the root tip meristematic tissue (Table 2a). Taken together, the results indicate that dhurrin pathway genes involved in biosynthesis, bio-activation and detoxification/recycling are regulated in very different ways during nodal root development although all pathway genes are highly expressed in nodal root tip-growing zones.

**Table 2:**
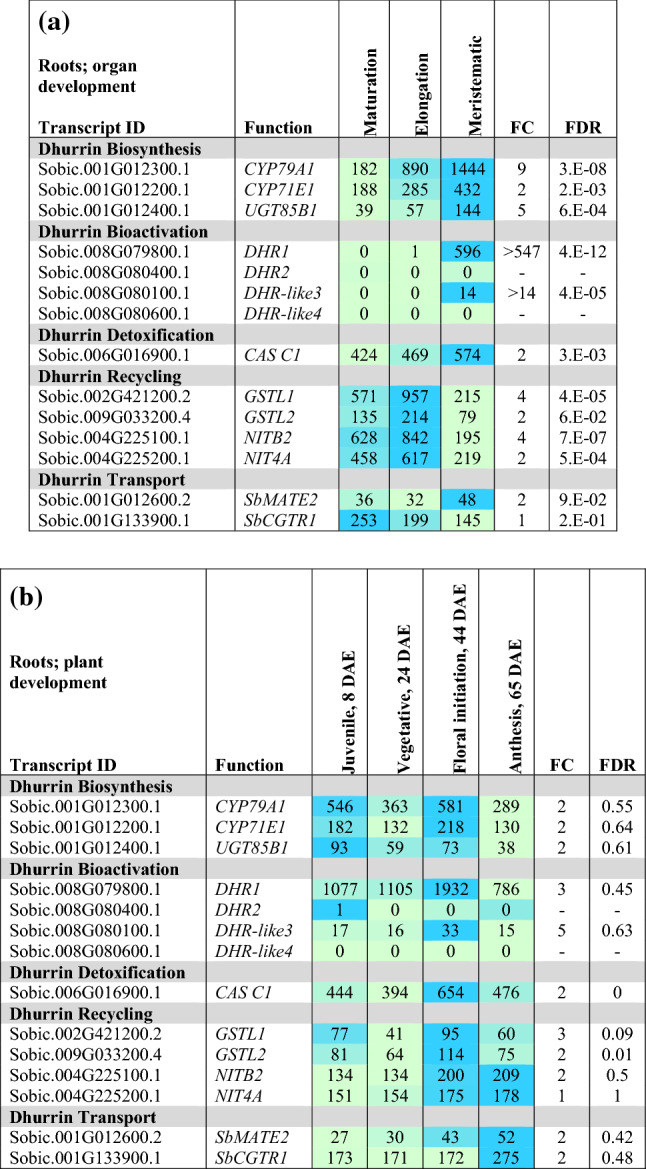
Heat map showing the expression (transcripts per million reads, TPM) of key cyanogenesis-related genes in root tissue of *S. bicolor*

(**a**) Three different specific zones of cell development in nodal roots of *S. bicolor* TX08001, indicated by green boxes in Fig. 2, *n* = 3, FC and FDR from edgeR. (**b**) Roots of *S. bicolor* BTx623 sampled at four different stages of plant development indicated by the yellow boxes in Fig. 2, *n* = 3, FC and FDR from edgeR. Root tissues with highest expression are shown in blue and lowest expression in green. Only data for genes with variation in expression are presented. Expanded tables that include genes with low or undetectable expression can be found in the Supplementary Information (Table S2)

Expression of genes in the dhurrin pathway was examined in roots of *S. bicolor* BTx623 at four stages of plant development (Table 2b; Fig. 2). At all stages of plant development, roots were collected from the portion of the soil profile where root proliferation was occurring as indicated by the presence of root tips. Expression of *CYP79A1, CYP71E1* and *UGT85B1* was high at all stages of plant development indicating that dhurrin biosynthesis occurs in proliferating root systems throughout plant development. *DHR1* was expressed at very high levels and *DHR-like3* at low levels throughout development whereas *DHR2* and *DHR-like4* expression was very low or not detected. In general, expression of genes involved in dhurrin biosynthesis, detoxification/recycling and transport was high in proliferating roots throughout plant development (Table 2b).

#### Expression of dhurrin pathway genes during stem development

Dhurrin pathway gene expression during early stages of stem internode development in the vegetative phase of R.07020 is shown in Table 3a. Nascent internodes of R.07020 produced by the shoot apical meristem increase in size through cell division, followed by a phase of elongation, growth cessation, and further differentiation (Kebrom et al. 2017). These four stages of internode development occur sequentially in the top four visible internodes of R.07020 (Table 3a, see Table S3a for a diagram of internode development). Expression of genes in the dhurrin pathway in the top four visible internodes was characterized to better understand dhurrin pathway gene expression during stem and internode development. *CYP79A1* and *CYP71E1* expression was high in internode 1 and 2, followed by a decrease in expression of ~ 2–3-fold in internodes 3 and 4. *UGT85B1* and *POR* expression was high and uniform in internodes 1–4 (Table 3a, Supplementary Table S3a). Among the dhurrinase genes, *DHR1* was highly expressed in internode 1 followed by a ~ 50-fold decrease in expression during this phase of internode development. *DHR-like3* expression was also highest in internode 1 and decreased steadily to ~ 100-fold lower levels in internode 4. *DHR2* was expressed at relatively low levels that did not change with development and *DHR-like4* expression was very low or undetectable in developing R.07020 internodes (Table 3a). *HNL* expression was low in all tissue sampled (Supplementary Table S3a), whilst *CAS C1* showed high and uniform expression across all internodes (Table 3a). *GSTL1* and *NITB2* expression increased markedly with internode development (Table 3a), whilst the expression of *GSTL2* was uniformly high (Supplementary Table S3a). Expression of the putative transporter *SbCGTR1* increased approximately 17-fold during this phase of internode development.

**Table 3:**
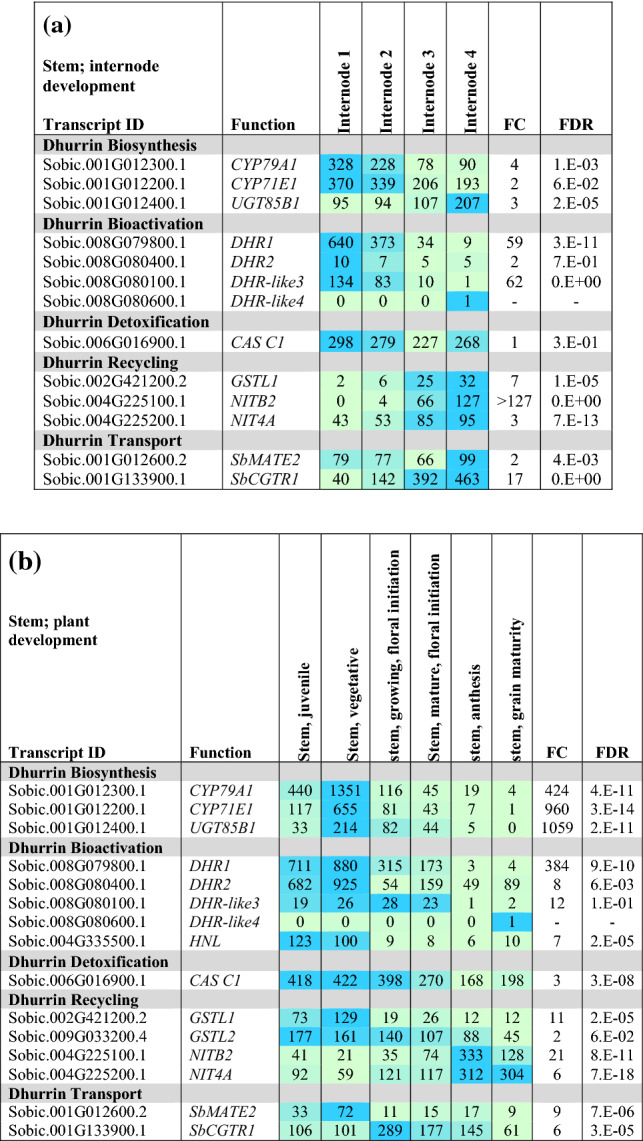
Heat map showing the expression (transcripts per million reads, TPM) of key cyanogenesis-related genes in true stem tissue (i.e. outer leaf sheaths removed) of *S. bicolor*

(**a**) The first four internodes from the shoot apex of *S. bicolor* R.07020 at 60 DAE. Internodes were numbered from the sub-apical internodes 1–4 below the apical dome (see Table S3b for details). (**b**) *S. bicolor* BTx623 at different stages of plant development (location indicated by red boxes in Fig. 2): 2 mm juvenile stem at 8 DAE entire stem; 1 cm vegetative stem at 24 DAE entire stem; Floral initiation (44 DAE); 2 cm long growing internode near apex; mature internode from middle of the stem; Internode; mid-stem anthesis (65 DAE); Internode, mid-stem grain maturity (96 DAE). Expression is given in Transcripts Per Million (TPM), *n* = 3, FC FDR from edge R. Only data for genes with changes in expression are presented. Expanded tables that include genes with low or undetectable expression can be found in the Supplementary Information (Table S3)

Expression of dhurrin pathway genes in stems during plant development was examined using stem tissues derived from BTx623 (Table 3b; Fig. 2 red boxes). The stems of juvenile-phase seedlings were ~ 2 mm in length 8 d after emergence (8 DAE) and 1 cm in length at 24 DAE, a short time after transition from the juvenile to the vegetative phase. Genes encoding the first three steps in dhurrin biosynthesis (*CYP79A1, CYP71E1, UGT85B1*) were expressed at relatively high levels in stems of juvenile seedlings (8, 24 DAE) prior to the onset of internode elongation (Table 3b). Expressions of dhurrin pathway genes were also analysed in stem internodes that were elongating at floral initiation and in non-growing internodes at anthesis and grain maturity. *CYP79A1, CYP71E1* and *UGT85B1* transcript levels were lower in elongating and fully elongated internodes of plants at floral initiation and still lower at anthesis or grain maturity (Table 3b). *POR* was expressed at similar levels in stems at all stages of plant development (Supplementary information Table S3b). *DHR1* and *DHR2* were expressed at high levels in growing stems of juvenile and vegetative plants. Expression levels of both dhurrinase genes were lower at later stages of plant development (Table 3b). *DHR-like3* was expressed at a low but constant level in stems through to floral initiation, whereas *DHR-like4* expression in stems was consistently low or not detected (Table 3b). Expression of *HNL*, *CAS C1, GSTL1 and GSTL2* was also highest in juvenile tissue and decreased with plant development, in a pattern similar to the dhurrinase genes. In the recycling pathway, *NIT4A/4B2* expression increased during plant development with maximum expression in plant stems at anthesis (65 DAE). Expression of the *SbMATE2* dhurrin transporter was highest in stems of juvenile and vegetative plants and ~ fourfold lower at later stages of plant development. The *SbCGTR1* putative transporter showed maximum expression in the stems at floral initiation (44 DAE).

#### Expression of dhurrin pathway genes in leaf sheaths

Information on dhurrin pathway gene expression during leaf sheath development was collected from elongating and fully elongated leaf sheaths of TX08001 during vegetative development. Variation in expression at later stages of plant development was obtained by analysis of fully expanded leaf sheaths of BTx623 at floral initiation, anthesis and grain maturity (Table 4; Fig. 2). *CYP79A1*, *CYP71E1* and *UGT85B1* were expressed at relatively high levels in growing leaf sheaths of TX08001. However, expression of these genes was reduced to low levels in fully expanded leaf sheaths of TX08001 and non-growing leaf sheaths of BTx623 at floral initiation, anthesis, and grain maturity. *POR* expression showed only small differences in expression during leaf sheath and plant development (Supplementary information Table S4). Expression of *DHR1* and *DHR2* was very low in all leaf sheath tissue sampled and *DHR-like3* was expressed at a low level in growing leaf sheaths of TX08001 (Table 4). However, expression of *DHR-like4* was high and expression of *HNL* was very high in the growing sheath of TX08001, decreasing ~ 50-fold in expanded sheath tissue of TX08001 (Table 4). In BTx623, *HNL* expression was highest in leaf sheath tissue at floral initiation followed by 54-fold lower expression at grain maturity. *CAS C1* expression in both TX08001 and BTx623 increased during leaf sheath development followed by a decrease in BTx623 at grain maturity. The *GSTL1* and *GSTL2* genes, involved in dhurrin recycling, were highly expressed in the growing sheath of TX08001, but at lower levels in BTx623 leaf sheaths post floral initiation. *NIT4A*, *NIT4B1* and *NIT4B2* were expressed in all leaf sheath tissues with up to fivefold variation depending on stage of plant development. *SbMATE2* dhurrin transporter gene expression was also relatively high in growing leaf sheaths of TX08001, lower in fully expanded leaf sheath tissue, and very low in BTx623 from floral initiation through to grain maturity. The *SbCGTR1* putative transporter is highly expressed in leaf sheath tissue with ~ threefold variation in expression during development (Table 4).

**Table 4:**
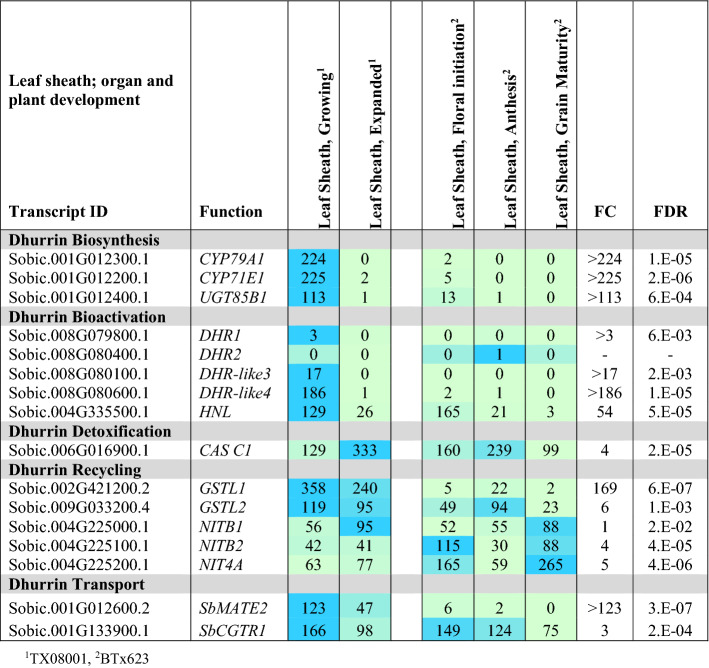
Gene expression (TPM) in leaf sheath tissue of two *S. bicolor* cultivars, TX08001 and BTx623, at different stages of plant development

Leaf sheath is defined as the outer leaf blades surrounding the internode stem sections. Sampled tissue is indicated by yellow circles in Fig. 2. Transcripts per million reads (TPM), *n* = 3, FC and FDR from edgeR. Only data for genes with changes in expression are presented. Expanded tables that include genes with low or undetectable expression can be found in the Supplementary Information (Table S4)

#### Expression of dhurrin pathway genes in leaves during plant development

Expression of dhurrin pathway genes was quantified in green leaf blades collected from BTx623 during the juvenile phase (8 DAE), early vegetative phase (24 DAE), at floral initiation, anthesis and at grain maturity. The 24 DAE RNA was extracted from a bulk sample of expanded leaf blades and growing leaves located in the whorl. *CYP79A1*,* CYP71E1* and *UGT85B1* expression was highest in leaves collected from plants at 8 and 24 DAE and very low in fully expanded leaves of plants at anthesis and grain maturity (Table 5). *POR* was expressed at relatively high levels in leaves until anthesis and at a lower level at grain maturity (Supplementary Table S5). *DHR2* was expressed at very high levels in leaf blades except at grain maturity. *DHR1* and *DHR-like3* expression was very low in leaf blades (Table 5). *DHR-like4* showed low levels of expression that increased to a peak at anthesis. *HNL* was expressed in young juvenile and vegetative-phase leaf blades then decreased to undetected levels at anthesis and grain maturity. *CAS C1* increased in expression up to the floral initiation stage, then decreased to approximately half in leaves of plants at grain maturity. In the endogenous recycling pathway, the *GSTL2* expression was approximately twice that of *GSTL1*, with both genes expressed at higher but uniform levels up to the floral initiation stage then decreasing to low levels at grain maturity (Table 5). Expression of *NIT4A*/*4B2* was high in leaf tissues with maximal expression at grain maturity, suggesting recycling may be higher at that stage of plant development to aid remobilization of stored N and C. The *SbMATE2* dhurrin transporter gene was expressed at low levels in leaves at all stages of development and very low levels at anthesis and grain maturity (Supplementary Table S5). Expression of the *SbMATE2* genes in leaves was also low relative to the expression of this gene in stems and roots. The *SbCGTR1* putative transporter gene also showed relatively low expression in leaves with peak expression in vegetative-phase leaves.

**Table 5:**
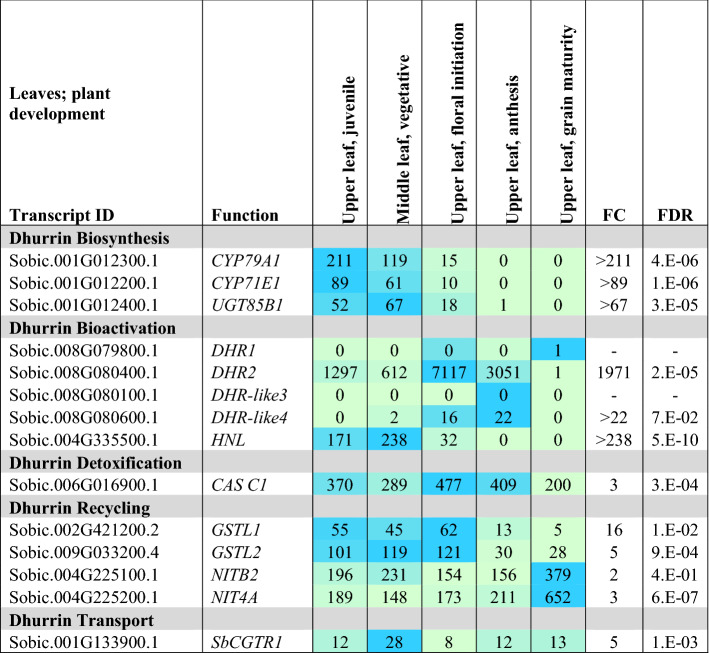
Heat map showing gene expression in leaves during *S. bicolor* BTx623 development, (denoted by blue circles in Fig. 2)

Juvenile, upper portion (distal) of third emerging leaf blade 8 DAE; vegetative, middle portion of shoot after removing first through fourth emerged leaves 24 DAE; floral initiation, upper portion (proximal) of leaf blade of last fully emerged leaf (ligulated) 44 DAE; anthesis, upper portion (distal) of leaf blade of leaf below flag leaf 65 DAE; grain maturity, upper leaf 96 DAE. TPM, *n* = 3, FC and FDR from edgeR. Only data for genes with changes in expression are presented. Expanded tables that include genes with low or undetectable expression can be found in the Supplementary Information (Table S5)

### Expression of dhurrin pathway genes in a developing phytomer of field grown TX08001

The expression of dhurrin pathway genes in TX08001 under field conditions was investigated 60 days after plant emergence during vegetative-phase growth (Table 6). Dhurrin pathway expression was examined in tissues collected from a single phytomer that contained a fully expanded leaf blade/sheath and a partially elongated internode. Expression of genes involved in dhurrin biosynthesis was high in the leaf sheath collar, but much lower in the leaf blade. In the leaf sheath, these genes were expressed at high levels in the sheath collar and base and lower in the middle of the leaf sheath. Expression of genes involved in dhurrin biosynthesis was highest in the pulvinus and basal growing zone tissues of the internode and lower in the nodal plexus. Expression of genes encoding *DHR1* and *DHR-like3* was low in leaf blades and sheaths, low in the nodal plexus and upper portions of the internode, with higher expression in the lower tissues of the internode and pulvinus. *DHR2* expression was low in most tissues of the phytomer. *DHR-like4* expression was high in the leaf collar and very low in all other tissues of the phytomer. Expression of *HNL* was high in the leaf blade, very high in the leaf sheath and very low in stem tissues.

**Table 6:**
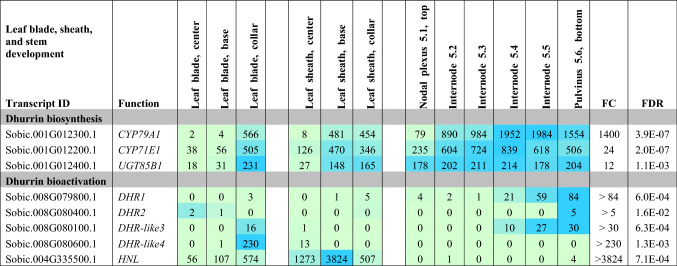
Heat map showing dhurrin pathway gene expression in the leaf blade, leaf sheath, and node–internode of one phytomer of *S. bicolor* TX08001 grown in the field for 60 days

The phytomer contained a fully expanded leaf/leaf sheath and an internode that was partially elongated. Leaf blade and leaf sheath samples were collected from the middle, base and collar of each organ. Stem samples were collected from the nodal plexus (5.1) where the leaf sheath joins the stem, internode (5.2–5.5, top to base) and pulvinus (5.5). TPM, *n* = 3, FC and FDR from edgeR. Data for genes involved in dhurrin biosynthesis and bioactivation are shown

### Coordinated expression of genes encoding dhurrinase and dhurrin biosynthesis

Three genes involved in dhurrin biosynthesis are clustered on chromosome 1 (*CYP79A1, CYP71E1, UGT85B1*) and genes encoding dhurrinases are clustered on chromosome 8 (Table 7b. The three genes involved in dhurrin biosynthesis have been previously reported to be co-expressed and co-expression was also observed in this study (Table 7b). Co-expression analysis of *DHR*-gene expression showed that *DHR1* and *DHR-like3* are also co-expressed (correlation = 0.91). *DHR1* and *DHR-like3* are differentially expressed in roots and stems and show higher expression in growing zones compared to older non-growing tissues (Table 7a). However, the ratio of expression of *DHR1/DHR-like3* can vary from ~ 42 in the root growing zone to ~ 1.8 in the stem internode growing zone indicating that *DHR-like3* plays a more significant role in stem growing zones and *DHR1* in roots (Table 7a). In contrast, *DHR2* expression is not correlated with other *DHR *genes with highest expression in tiller buds, mature leaf blades and lower expression in the stem apex, possibly associated with nascent leaf formation (Table 7a, b). High *DHR2* expression was observed in vegetative-phase young leaves of BTx623 grown in growth chambers (~ 2861 TPM), however, *DHR2* expression was low in leaves of TX08001 grown in the field (2 TPM) (Table 7a). The basis of this difference is unclear but may be due to differences in stage of leaf development, genotype, and environmental factors. *DHR-like4* expression was also highly specific to the leaf collar of young leaves and low expression in the upper portion of the leaf sheath (Table 7a). Genes involved in dhurrin biosynthesis and dhurrinases were often co-expressed in organ growing zones, but not in fully developed organs (Table 7b). For example, in fully elongated non-growing (NG) stem internodes and roots, genes involved in dhurrin biosynthesis were highly expressed when genes encoding dhurrinases were expressed at very low levels (Table 7b). In contrast, expression of *DHR2* was very high in non-growing leaves of BTx623 at anthesis although expression of genes involved in dhurrin biosynthesis was very low. This temporal difference in expression could help separate synthesis of dhurrin and proteins that are involved in bio-activation, or it could reflect other non-defence functions associated with DHR2.

**Table 7:**
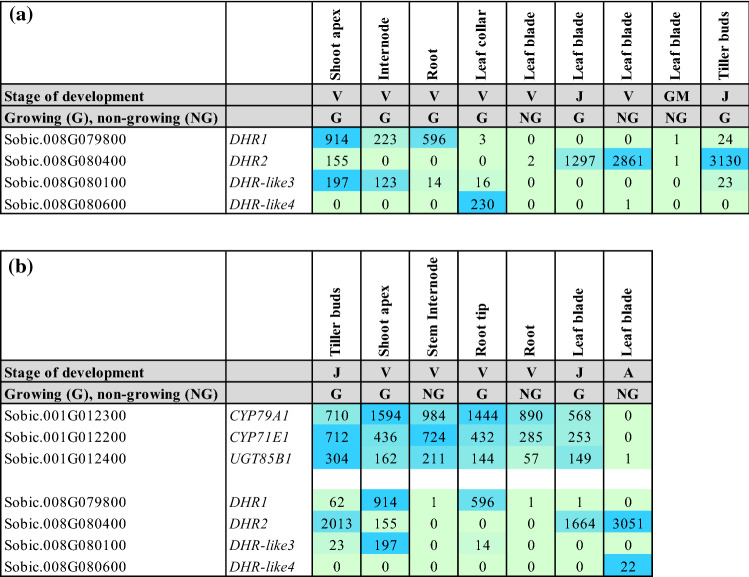
Heat map showing dhurrin pathway gene expression in *S. bicolor* organs, stages of organ development and plant development

(**a**) Differential expression of genes that encode dhurrinase in organs and stages of growth/development. (**b**) Differential expression of genes involved in dhurrin biosynthesis and genes encoding dhurrinase in *S. bicolor* in different organs (tiller buds, shoot apex, stem internode, roots, leaf blade), growing (G) and non-growing tissues (NG), and stages of plant development (juvenile (J), adult vegetative (V), anthesis (A)). TPM, *n* = 3, FC and FDR from edgeR. Data for genes involved in dhurrin biosynthesis and bioactivation are shown

#### HCNp and β-glucosidase activity in tissues of roots, shoots and tiller buds

Hydrogen cyanide potential (HCNp) in nodal roots of BTx623 equivalent to the stages in Table 2a was highest in the portion of the root that spans the growing zone and lower in the fully elongated root tissue (Fig. 3). We also measured HCNp in young developing leaves and older fully developed leaves of BTx623 at or shortly after floral initiation. To confirm the activity of *DHR2* in these leaves, we compared the amount of hydrogen cyanide released with and without the addition of exogenous β-glucosidase in four individual plants. Greater amounts (~ two-fold) of evolved HCN were observed upon addition of exogenous β-glucosidase. The analysis showed higher HCN production in young growing leaves in plants 2, 3 and 4 compared to older fully expanded leaves (Fig. 4).

### Expression of dhurrin pathway genes in leaves during a diel cycle

Expression of dhurrin pathway genes in young leaves during a diel cycle was investigated in vegetative-phase BTx623 plants. Tissue sampling started within 10 min after dawn (time 0) and samples were collected every 3 h, including a 10-h night period and four time points into the light period of day 2. Transcript abundance of biosynthesis pathway genes *CYP79A1, CYP71E1* and *UGT85B1* peaked in the morning (3HL and 27HL) and 2 h after the onset of dark (15HL) (Fig. 5). In the morning, *UGT85B1* expression was lower than *CYP79A1* and *CYP71E1*, but in the evening, *UGT85B1* expression was similar to or higher than *CYP79A1/CYP71E1*. *POR* expression varied ~ 50-fold during a diel cycle with highest expression at dawn followed by a decrease in transcript levels to low levels from 9HL until the following dawn. *DHR2* transcript levels were high throughout the day–night cycle. By contrast, expression of *HNL* was highest during the day peaking at 9HL followed by a decrease in the late afternoon and at night (Fig. 5). *NIT4B2* expression increased at dawn and stayed high for the first 6 h of the day then decreased in the evening. Expression of the *SbMATE2* dhurrin transporter increased in the morning and remained high during the day and for 1 h after darkness, then decreased to low levels for the duration of the night, increasing the following dawn.

## Discussion

A detailed understanding of gene expression in plant organs/tissues during development and in response to variation in nutrient status and environmental constraints facilitates the identification of signalling pathways, transcription factors and gene regulatory networks that modulate gene expression. In this study, RNA-seq was used to characterize the expression of genes involved in the biosynthesis, recycling and bio-activation of dhurrin during organ and plant development in sorghum. Broadly, we found that expression of genes involved in dhurrin biosynthesis was highest in growing tiller buds, imbibed seed, and the growing zones of leaves, roots, and stems. The bio-activation genes showed organ- and tissue-specific expression whereas expression of the recycling genes was maintained or increased during the reproductive phase. The expression of genes involved in dhurrin biosynthesis and recycling in leaves varied during diel cycles. If the function of dhurrin is for herbivore defence, then synthesis and bio-activation would be expected to vary with the stage of development and within different tissues, with the highest deployment in juvenile or meristematic tissues following a typical ontological defence trajectory (Boege and Marquis 2005). The expression of genes in the dhurrin pathway was complex and varied with plant development in a way that is consistent with multiple roles in defence and metabolism, including serving as a source of reduced nitrogen during grain filling and germination. Instances of differential expression of biosynthesis and bio-activation genes, and the tissue-specific expression of *DHR1*, *DHR2* and *DHR-like4* indicates the involvement of complex regulatory systems. If the multiple and changing roles for dhurrin over the life of the sorghum plant—herbivore defence, mitigation of abiotic stresses, nitrogen storage and recycling—are found to be a general feature of other cyanogenic plants, this may explain why cyanogenic plants are so common, and why humans appear to have selected for higher expression of this pathway during domestication.

### High transcript levels of biosynthesis genes in young tissues indicate a defence function

The three genes involved in dhurrin biosynthesis that are clustered on chromosome 1 (*CYP79A1, CYPE1, UGT85B1*) are generally co-expressed in developing organs and during plant development. These genes were highly expressed in growing zones of leaves, stems (shoot apex, base of elongating internodes), roots and young developing tiller buds and imbibing seeds. Expression extended into zones of elongation and early maturation of developing roots and stem internodes with gradual decreases in expression of these genes as the organ or tissue became fully differentiated. High expression was also observed in leaf collars and the stem pulvinus, tissues adjacent to elongating leaf blades and stem internodes. These tissues may be prioritized for protection or sources of dhurrin transported into developing leaf blades and internodes. High rate of dhurrin synthesis in young developing organs and growing zones is consistent with elevated priority of deployment of protection. Very low expression of genes involved in dhurrin biosynthesis was observed in older fully developed organs of vegetative-phase adult plants (i.e., leaf blades of TX08001 in the field) and during the reproductive phase, especially from anthesis and grain maturity (i.e., leaf blades, leaf sheath and stems of BTx623). Down-regulation of dhurrin biosynthesis in vegetative organs during the reproductive phase of development would reduce competition for nitrogen needed for grain filling.

Previous studies have consistently found young sorghum plants and immature tissues of older plants to have much higher concentrations of cyanogenic glucosides (Halkier and Møller 1989; Miller et al. 2014; Blomstedt et al. 2018; Sohail et al. 2020; Cowan et al. 2021). Here we determined that the HCNp in roots was highest in a region immediately adjacent to the root tip that spans the growing zone, with a significantly lower concentration in the older tissue of the maturation zone (Table 2b; Fig. 3). During root development, transcript levels were correlated with accumulation of dhurrin (Fig. 3), supporting the earlier observation of Busk and Møller (2002) that dhurrin biosynthesis is transcriptionally regulated.

In leaf blades, genes involved in dhurrin biosynthesis are also expressed at higher levels when they are growing (Table 5). Dhurrin concentrations in the leaves are significant in older leaves (Fig. 4), even though expression of genes involved in de novo synthesis is very low in these older tissues (Tables 5, 7a). This is likely due to the presence of stably stored dhurrin synthesized during leaf development (Knudsen et al. 2020) or could be due transport of dhurrin into fully developed portions of leaf blades from the base of the leaf blade or leaf collars (see below). Similar expression patterns of genes encoding biosynthesis of the cyanogenic glucoside linamarin in the rubber tree, *Hevea brasiliensis* were obtained upon analysis of transcriptome prepared from leaves of different age classes (Fang et al. 2016).

The first step in the biosynthesis pathway, the conversion of tyrosine to *(E)-p*-hydroxyphenylacetaldoxime is catalysed by CYP79A1 and is considered a rate limiting step (Busk and Møller 2002). However, if this step proceeds faster than the subsequent formation of *p*-hydroxymandelonitrile catalysed by CYP71E1, then the intermediate E-oxime would accumulate. Oximes with the E-configuration have high biological activity compared with Z-oximes exhibiting strong insecticidal, fungicidal, and herbicidal activity (Sørensen et al. 2018). They inhibit mitochondrial oxidases and thereby promote lipid peroxidation and the production of toxic reactive oxygen species (Sakurada et al. 2009; Møller 2010). Upon introduction of CYP79A1 and CYP71E1 into *Arabidopsis*, the plants do not develop properly due to auto-toxicity related to *p*-hydroxymandelonitrile accumulation or the associated liberation of hydrogen cyanide. Upon introduction of the entire dhurrin pathway, the glucosylating activity of the introduced UGT85B1 efficiently converts *p*-hydroxymandelonitrile into dhurrin and the wild-type phenotype is restored (Tattersall et al. 2001b). In the transcriptomes here analysed, we observe co-expression of *CYP79A1*, *CYP71E1* and *UGT85B1* in a tissue although not always at equal transcript levels at the time of day the tissue was collected. In leaves, *CYP79A1* and *CYP71E1* were more highly expressed than *UGT85B1* in the morning when most samples were collected, whereas *UGT85B1* expression was higher in the evening. A similar pattern is seen for equivalent genes in barley (Knoch et al. 2016).

### Bio-activation: tissue-specific dhurrinase and HNL expression allow HCN release to be finely regulation

Phylogenetic analysis of *DHR1* and *DHR2* identified two additional genes that encoded closely related proteins we named *DHR-like3* and *DHR-like4*. All four genes are located in close proximity on sorghum chromosome 8. In our current study, we characterized the expression of *DHR1*,* DHR2* and the two *DHR-*like genes (Table 7a, b). Follow-up studies will be required to determine if DHR-like3 and DHR-like4 catalyse dhurrin hydrolysis. Ganjewala et al. (2010) noted that expression of *DHRs* is often localized to specific organs. Here, we confirm that the two previously identified *DHRs* (*DHR1* and *DHR2*) show a high degree of tissue specificity with *DHR1* differentially expressed in roots > shoot apex/stem intercalary meristem/pulvinus and *DHR2* in leaf blades (Tables 2, 5, 6). *DHR1* is highly expressed in imbibed seeds undergoing root outgrowth and *DHR2* is highly expressed in developing tiller buds associated with nascent leaf growth. Both *DHR1* and *DHR2* are highly expressed in stem tissue that includes the shoot apex of juvenile and vegetative stems (24 DAE) that have not started to elongate (Table 3a). Krothapalli et al. (2013) also found that *DHR1* was differentially expressed in root tissue and the mesocotyl. *DHR2* was expressed at high levels in young developing leaves and fully developed leaves (anthesis) of BTx623 grown in growth chambers/greenhouse (Table 5). However, *DHR2* expression in leaves of field grown TX08001 was much lower indicating growing conditions modulate *DHR2* expression (Table 6). Overall, expression of the dhurrinase-like genes *DHR-like3* and *DHR-like4* was low relative to *DHR1* and *DHR2*, with two notable exceptions: *DHR-like3* was expressed in the stem shoot apex and stem intercalary meristematic regions (Tables 3, 6) and *DHR-like4* was highly expressed in leaf collars with lower expression in upper portion of leaf sheaths of the bioenergy cultivar TX08001 (Tables 4, 6). *DHR1* and *DHR-like3* expression was correlated, although *DHR1* is more highly expressed in roots and *DHR-like3* showed highest expression in the shoot apex and stem intercalary meristems. These genes are adjacent on chromosome 8, possibly the result of a recent gene duplication. It is interesting that expression of *DHR-like4* is highly expressed in leaf collars and not correlated with the expression of any of the other members of this gene family. This is typical of gene paralogs that diverge and acquire unique patterns of expression that in this case may help protect the leaf collar. *DHR1*,* DHR2* and *HNL* were expressed at high levels in young tissue (roots, stem, sheath, tiller buds), decreasing with plant age (Tables 2, 3, 4; Table S1). *DHR-like3* expression also decreased with organ maturity, consistent with our observations that dhurrin metabolism is associated with early organ growth and development. Follow-up studies will be required to confirm that *DHR-like3* and *DHR-like4* encode dhurrinases and to better understand the activity of these enzymes.

Many cyanogenic species encode two or more dhurrin β-glucosidase isoenzymes (Hösel et al. 1987). The genome of *Lotus japonicus* harbours four genes encoding β-glucosidases able to hydrolyze aliphatic cyanogenic glucosides. These four genes are differentially expressed; BGD2 and BGD4 are expressed in the shoot, BGD3 in the keel and the enclosed reproductive organs of the flowers, whilst BGD7 is expressed in roots (Morant et al. 2008a; Lai et al. 2014; Takos et al. 2011). The confined expression of the individual dhurrinases to specific tissues observed here may be related to the presence of particular cell types in the relevant organs. Kojima et al. (1979), for example, found the DHR in leaves of sorghum to be specific to mesophyll cells, and possibly even the chloroplast (Kojima et al. 1979; Thayer and Conn 1981). Elevated expression of dhurrinase could occur in the root cortex requiring root cortical cell specific expression. RAMAN, Laser dissection/qPCR, in situ hybridization and micro-extraction technologies will be useful for defining where root dhurrinase is transcribed and accumulates (Heraud et al. 2018; Montini et al. 2020).

Upon cell disruption dhurrin is hydrolysed by dhurrinases (β–glucosidases) and hydrogen cyanide is rapidly released from dhurrin through the action of α-hydroxynitrilases (HNLs) with co-production of stoichiometric amounts of *p-*hydroxybenzaldehyde. High expression of *HNL* is leaf blades and the leaf sheath is consistent with a role of this enzyme in defence since these organs are exposed to pests. In contrast, *HNL* expression was low in stems even though genes involved in dhurrin biosynthesis were expressed at high levels. One could posit that since the stem is enclosed by the leaf sheath that has high expression of *HNL*, there is less need for rapid *HNL*-mediated release of HCN by stem tissue. Low HNL levels in stems may increase flux through the recycling pathway or facilitate dhurrin transport without turnover.

Cyanogenic glucosides are usually considered to be phytoanticipins, that is, they are synthesised and stored and only release the toxic HCN when mixed with specific hydrolytic enzymes (β-glucosidase and α-hydroxynitrile lyase). The rate of this release is important for the effectiveness of the system in deterring herbivores (Krothapalli et al. 2013). Ballhorn et al. (2006), for example, found a strong correlation between the activity of β-glucosidase and deterrence of herbivores in lima bean (*Phaseolus lunatus*). One might expect synchronous expression of biosynthesis and bio-activation genes, even though they are clustered on two different chromosomes as found by Fang et al. (2016) for *Hevea brasiliensis*. High expression of genes involved in dhurrin biosynthesis and organ-specific DHRs was observed in regions of organ cell division (i.e., root tips, shoot/stem apex, internode 1, intercalary meristems). However, there were notable exceptions, such as high *DHR2* expression in fully expanded leaf blades despite biosynthetic gene expression decreasing to very low levels (Tables 5, 7). Similarly, *DHR1* expression decreased to low levels in root tissues just above the root tip whereas expression of the three genes involved in dhurrin biosynthesis remained elevated. This difference may allow plants to restrict synthesis of *DHR1* to specific cell types generated in the meristematic zone, but continue to accumulate dhurrin for sequestration in vacuoles in elongating and fully elongated tissues. These differences in the timing of gene expression during organ biogenesis will need to be accounted for in gene regulatory network analysis.

The transport of specialized metabolites, such as dhurrin, is important to enable optimisation of the production costs and to ensure the compounds are deposited where they are most useful. MATE and NPF transporters have been implicated in relocation of various secondary metabolites, including dhurrin, linamarin and glucosinolates (Darbani et al. 2016; Nour-Eldin et al. 2012; Jørgensen et al. 2017). In our study, expression of *SbMATE2* increased in imbibing seed and decreased > tenfold in the leaf sheath post floral initiation. However, in most other organs and stages of development expression of this gene was relatively high and consistent. Darbani et al. (2016) showed that *SbMATE2* specifically transports dhurrin across the vacuolar membrane and it may be that *Sb*MATE2 is involved in the transport of dhurrin for defence and remobilization depending on the stage of organ development. We also analysed the expression of *SbCGTR1*, a NPF transporter identified based on its high (72%) amino acid sequence similarity to *At*GTR1 of *Arabidopsis* (Nour-Eldin et al. 2012) and 75% sequence similarity to *Me*CGTR1 of cassava (Jørgensen et al. 2017). The expression of *SbCGTR1* increases with internode development but otherwise was similar in organs and stages of plant development. Regulation of transport and re-allocation of dhurrin between tissue types in preparation for remobilisation and/or recycling of reduced nitrogen at later developmental stages could occur at a post-transcriptional level.

### Increase in recycling genes in maturing plants and with shift to reproductive stage is consistent with dhurrin as a mobilizable source of nitrogen

As the sorghum plants transitioned into the reproductive phase, a substantial decrease in the expression of the biosynthetic genes was observed concomitant with relatively small but consistent increases in expression of selective recycling genes. For example, *NITB2* expression increased ~ 127-fold during vegetative internode development (Table 3a) and increased ~ 21-fold in stems during the transition from the vegetative phase to anthesis (Table 3b). Potentially, this could orchestrate the mobilization of dhurrin-bound nitrogen for plant growth or to support seed production (Tables 2a, 4, 5). The high expression of genes involved in dhurrin biosynthesis in stems during internode biogenesis, low expression of *HNL* in internodes, and up-regulation of *NITB2* in stems post-floral initiation are consistent with dhurrin remobilization. It is clear that the recycling pathway, where dhurrin is metabolized without the release of toxic hydrogen cyanide affords the possibility of repurposing cyanogenic glucosides (Bjarnholt et al 2018). The expression of recycling genes, particularly around floral initiation, anthesis and grain maturity, may reflect the increased demand for nitrogen for seed set and filling. Root tissue just above root tips had a high dhurrin content although lower content in older more mature root tissue. The same root zone showed high expression of genes involved in dhurrin recycling, suggesting that the dhurrin present was subject to continuous endogenous recycling to provide nitrogen and carbon for growth uses (Fig. 4; Table 2b). In the rubber tree (*Hevea brasiliensis*), cyanogenic glucosides have been reported as being a mobilizable source of nitrogen and glucose used to build up the biosynthetic machinery required for latex production (Kongsawadworakul et al. 2009). The increased expression of *CAS* and *NIT4A* in mature rubber leaves observed by Fang et al. (2016) supports this hypothesis.

### Diel effects show dhurrin concentrations are environmentally plastic

We detected significant diel variation in the transcript levels of the biosynthesis genes. *CYP79A1* and *CYP71E1* showed a peak of expression 3 h after initiation of the light period concomitant with a small increase in *UGT85B1* expression. The transcript levels diminished during the light period followed by a small rise in expression about 1 h after onset of the dark period and a larger increase in *UGT85B1* expression. *POR* expression was highest at the beginning of the light period and the following 3 h whereas *HNL* expression peaked at 9HL and decreased at night. Expression of *DHR2* was high and showed only slight changes during diel cycles. Diel variation in gene expression can affect protein levels on a daily basis if protein stability is low, or more often, adjust levels of protein over a longer time frame. The oscillations may indicate signalling that connects expression of genes in the dhurrin pathway to the nitrogen demands of primary metabolism. Diurnal variation in synthesis of cyanogenic glucosides has previously been reported in cassava (Schmidt et al. 2018). In cassava, the transcripts encoding the biosynthetic genes disappeared at the beginning of the light period closely accompanied by disappearance of the biosynthetic enzymes and rapid recycling of the cyanogenic glucosides. It is possible that strong photosynthetic carbon fixation happening during the light period in the tropical and subtropical regions where cassava is grown may be balanced by a supply of reduced nitrogen in the form of ammonium ions derived from recycling of cyanogenic glucosides. The operation of a similar process in sorghum involving recycling of dhurrin is supported by the high transcript level of *HNL* and the *NIT4B2* during the light period. Like cassava, sorghum is grown in geographic regions with high light irradiation levels during the day. The recycling of dhurrin in the light period may serve to balance the nitrogen demands in primary metabolism when carbon fixation rates are high.

### Function and adaptive significance of cyanogenic glucosides

**Fig. 6:**
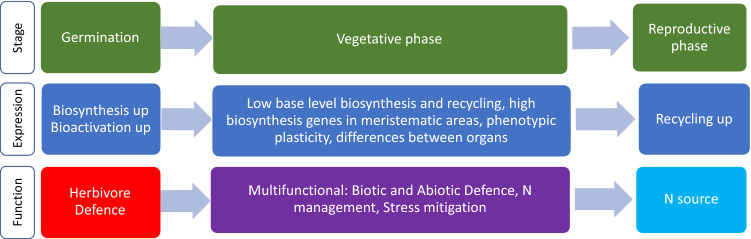
Model linking differences in gene expression of cyanogenesis-related genes at different stages of plant development with proposed functions of dhurrin. When plants are young, and in young tissues dhurrin is primarily functioning in herbivore defence. During vegetative growth, dhurrin has multiple roles mediated by environmental plasticity in response to light and abiotic stress. After anthesis, dhurrin becomes a source of reduced nitrogen to improve grain filling

Three distinct expression patterns of the cyanogenic glucoside biosynthetic pathway genes in sorghum were observed in this study: (1) high expression of biosynthesis genes in young developing tissues and organs; (2) a diel pattern in leaves, with enhanced expression of the biosynthetic genes in the period immediately following dawn; (3) an increase in cyanogenic glucoside recycling genes post floral initiation. These phases align with ontological development and are consistent with the hypothesis that cyanogenic glucosides serve multiple functions in plants—herbivore defence, mitigation of stress, UV scavengers, nitrogen storage and recycling—arising from different evolutionary pressures at different stages of plant development and in different tissues (Gleadow and Møller 2014). We have developed a schematic model linking expression levels, plant developmental stage and function (Fig. 6). Different patterns of expression in different tissues support the notion of multiple roles. In addition to the biosynthesis and recycling pathways, the presence of multiple nitrilases, GSTs and DHRs, some with tissue-specific expression, increases the options for additional flexibility in phenotypic expression in space and time.

High expression of bio-activation and biosynthetic genes, but not recycling genes, indicates a primary role in defence in the early stage of organ and plant development consistent with the very high concentration of dhurrin observed in these tissues and consistent with ontogenetic trajectories observed in sorghum (Miller et al. 2014; O'Donnell et al. 2013). This pattern supports the optimal allocation theory, which hypothesises that plants will invest resources in defence of the most valuable and most vulnerable parts of the plant (McKey 1974). Similar ontogenetic and spatial patterns have been observed in many species for a wide range of chemical defence systems (Barton and Boege 2017). As organs and plants mature (Phase 2), there is less value in investing in defence per se (Boege and Marquis 2005). In sorghum, such investments are plastic, with increased allocation of resources to dhurrin in plants experiencing stress (e.g. Rosati et al. 2019; O'Donnell et al. 2013; Gleadow et al. 2016)), or in the case presented here, transcript level variation guided by the diel cycle (Fig. 5). Such changes in expression that modulates pathway deployment are likely related to the availability of energy to reduce nitrogen in the leaves, or to mitigate stress by reducing reactive oxygen species. Once sorghum plants enter the reproductive phase (Phase 3), herbivore defence becomes less important and grain yield becomes the main evolutionary driver. In accordance with these demands, dhurrin accumulated in the early phases of grain development is re-cycled during the subsequent maturation period. As a result, only negligible residual dhurrin amounts are present in the mature grain (Nielsen et al. 2016). A similar link has been seen in *Nicotiana attenuata* (Diezel et al. 2011). Indeed, lack of nitrogen is an important factor in limiting grain growth in cereals, including sorghum. The decrease in expression of genes involved in dhurrin biosynthesis and an increase in the expression of recycling genes that we observed in plants during the reproductive phase supports the hypothesis that sorghum remobilises dhurrin to provide nitrogen for the developing grain. This could be one reason why stay green varieties of sorghum have higher levels of dhurrin (Hayes et al. 2015, 2016) and may help explain why domesticated sorghum is much more cyanogenic than its wild relatives (Myrans et al. 2021; Cowan et al. 2021).

A plant’s investment in the synthesis and maintenance of specialised metabolites is often quantified by calculating the costs in terms of growth forgone or reduced yield. While this approach is excellent at explaining why costs of defence change as plants mature (Boege and Marquis 2005), it does not account for the fact the costs are sometimes not detectable (e.g. (Sohail et al. 2020)). Results support the conjecture that dhurrin is not purely synthesised for defence but is integrated with the primary metabolism in sorghum functioning as a mobilizable source of reduced nitrogen during grain filling and germination. Allowing for roles other than defence can accommodate such discrepancies, at least for cyanogenic glucosides, while leaving the general tenant of resource allocation theory intact (Neilson et al. 2013). Clearly, different functions are of adaptive significance at different stages of development. Thus, rather than viewing the allocation of resources through the lens of ‘costs’ and ‘benefits’, it may be more useful to view them as integrated to the benefit of plant development and fecundity.

Any increase in nitrogen use efficiency would both increase yields and reduce costs to farmers. A significant amount of the nitrogenous fertiliser used in agriculture is currently wasted through either run-off or is converted to nitrous oxide, a potent greenhouse gas. Optimisation of dhurrin pathway gene expression could contribute to a reduced need for nitrogen fertilisers. The work presented here provides a number of options for doing this. While simply removing dhurrin biosynthesis seems an attractive option to reduce nitrogen use in cyanogenic plants, the observation that the storage of high-value reduced nitrogen that can be remobilized for grain yield may mean this is counterproductive, at least for grain crops. However, if the remobilization of nitrogen from dhurrin could be more targeted in space and time, this could lead to the desired improvements particularly in biomass production with a reduced need for fertilisers.

#### *Author contribution statement*

JM and RMG conceived of the project using RNA-seq data provided by JM. BMc performed the bioinformatics. All authors analysed and interpreted the results and contributed to the writing of the manuscript.

## Supplementary Information

Below is the link to the electronic supplementary material.

Supplementary file1 (DOCX 1044 KB)

Supplementary file2 (XLSX 59 KB)

## Data Availability

Expression data are available in the accompanying excel files. All of the raw data are available through Phytozome (Joint Genome Institute) https://genome.jgi.doe.gov/portal/.

## Abbreviation

HCNp: Hydrogen cyanide potential
