# Supplementary material for: Regulation of dhurrin pathway gene expression during Sorghum bicolor development
Source: Planta. 2021 Nov 11;254(6):119. doi: 10.1007/s00425-021-03774-2 (PMC8585852; doi:10.1007/s00425-021-03774-2)
Supplement: Supplementary file 1 — Supplementary file1 (DOCX 1044 KB) [file 425_2021_3774_MOESM1_ESM.docx]

**Regulation of dhurrin pathway gene expression during sorghum development**

**(Supporting information)**

**Running title: Transcripts of dhurrin metabolism genes in sorghum**

Roslyn M. Gleadow^1^, Brian A. McKinley^2^, Cecilia K. Blomstedt^1^, Austin C. Lamb^2^, Birger L. Møller^3^, John E. Mullet^2^

**Affiliations**

^1^School of Biological Sciences, Monash University, Vic, Australia

^2^Department of Plant Biochemistry and Biophysics, Texas A & M University, College Station, TX, USA

^3^Plant Biochemistry Laboratory, Department of Plant and Environmental Sciences, University of Copenhagen, Denmark

**Corresponding Author**

John Mullet;

jmullet@tamu.edu

## Supporting Information

**Differential expression of dhurrin metabolism genes shows defence and growth are entangled not alternatives**

Roslyn M. Gleadow, Brian McKinley, Cecilia K. Blomstedt, Austin Lamb, Birger Lindberg Møller, John Mullet

The following Supporting Information is available for this article:

**Fig. S1** Sequence alignment of four selected dhurrinase proteins from *S. bicolor*

**Fig. S2** Phylogenetic tree of genes containing the cytochrome P450 domain (PF0067) in *S. bicolor*

**Fig. S3** Phylogenetic tree of genes containing the glycoside hydrolase 1 domain (pf00232) in *S. bicolor*

**Fig. S4** Root sections collected from *S. bicolor* for assay of HCNp

**Tables S1-S6** Heat maps showing expression of all cyanogenesis related genes are included in Supporting Information as a separate excel file.

**Table S7**  Phytozome and GenBank gene identifiers.

**Methods S1** Basis for selection of genes for analysis (description)

**Fig. S1** Alignment of the proteins encoded by the four selected dhurrinase genes in *Sorghum bicolor*.

DHR2_Sobic.008G080400 MAPLLLHASAISHSAHP-GLRSHIG-PNNEHISRHLS-S-SSQNTKRRCNISLRSRAQRI 56

DHRlike4_Sobic.008G080600 --MALLLASAMNHSAHPAGLRSQ---SNNQSFSRHHLCSSPKNISKRRCKLSFRPRAERV 55

DHR1_Sobic.008G079800 --MALLLASAMNHTAHPAGLRSH---PNNESFSRHHLCSSPQNISKRRSNLSFRPRAQTI 55

DHRlike3_Sobic.008G080100 --MALLLASAMNHTAHPAELRSHLGRPNNESFSRHHLCSSPQNISKRRSNLSFRPRAQTI 58

** ***:.*:*** ***: **: :*** * .: :***.::*:* **: :

DHR2_Sobic.008G080400 SSQ-LGGQKLEHWEIPKRDWFPPSFTFGAATSAFQIEGGWNEDGKGPSTWDHFCHTYPDF 115

DHRlike4_Sobic.008G080600 GSE-NGNHRLSPREIPRKDWFPPSFLVSAATSAYHIEGAWNEDGKGPSTWDHFCHEYPER 114

DHR1_Sobic.008G079800 SSESAGIHRLSPWEIPRRDWFPPSFLFGAATSAYQIEGAWNEDGKGPSTWDHFCHNFPEW 115

DHRlike3_Sobic.008G080100 SSE-PGIHMLSPWEIPRRDWFPPSFLFGAATASYQIEGAWNEDGKGPSTWDHFCHNFPEW 117

.*: * : *. ***::******* ..***::::***.**************** :*:

DHR2_Sobic.008G080400 IADKSNGDVAADSYHLYEEDVKLLKEMGMDAYRFSISWPRILPNGTLS-DINEKGIAYYN 174

DHRlike4_Sobic.008G080600 IADRSNGDVAADSYHMYADDVKLLKEMGMDAYRFSISWSRILPKGTIAGGINEKGVEYYN 174

DHR1_Sobic.008G079800 IVDRSNGDVAADSYHMYAEDVRLLKEMGMDAYRFSISWPRILPKGTLAGGINEKGVEYYN 175

DHRlike3_Sobic.008G080100 IVDRSNGDVAADSYHMYAEDVRLLKEMGMDAYRFSISWPRILPKGTLAGGINEKGVEYYN 177

*.*:***********:* :**:**************** ****:**:: .*****: ***

DHR2_Sobic.008G080400 NLINLLIDNGIEPYVTIFHWDTPQALVDDYGGFLDKRIIKDYTDFAGLCFERFGDRVNNW 234

DHRlike4_Sobic.008G080600 KLIDLLLENGIEPYITIFHWDTPQALVDAYGGFLDDRIITDYTDFAKVCFQKFGTKVKNW 234

DHR1_Sobic.008G079800 KLIDLLLENGIEPYITIFHWDTPQALVDAYGGFLDERIIKDYTDFAKVCFEKFGKKVKNW 235

DHRlike3_Sobic.008G080100 KLIDLLLENGMEPYITIFHWDAPQALVDTYGGFLDERIIKDYTDFAKVCFENSGKKVKNW 237

:**:**::**:***:******:****** ******.***.****** :**:. * :*:**

DHR2_Sobic.008G080400 LTFNEPHTFTCLSYGTGILAPGRCSPGMKCPDPTGDSIREPYLVGHNFLLAHAETVDLYN 294

DHRlike4_Sobic.008G080600 FTFNEPETFCSVSYGTGVLAPGRCSPGVNCAVPTGNSLTEPYTVAHHLLLAHAETVDLYN 294

DHR1_Sobic.008G079800 LTFNEPETFCSVSYGTGVLAPGRCSPGVSCAVPTGNSLSEPYIVAHNLLRAHAETVDIYN 295

DHRlike3_Sobic.008G080100 FTFNEPETFCSVSYGTGVLAPGRCSPGVSCAVPTGNSLTEPYIVAHNLLRAHAETVDLYN 297

:*****.** .:*****:*********:.* ***:*: *** *.*::* *******:**

DHR2_Sobic.008G080400 KFHRGEKGRIGLALNVMGTVPYGSTFLDEQAHERCMDYNLGWYLEPVVRGDYPHSMRSSV 354

DHRlike4_Sobic.008G080600 KHHK-------------GRVPYTNTFLDQQAQERSMDNCLGWFLEPVVRGDYPFSMRASA 341

DHR1_Sobic.008G079800 KYHKGADGRIGLALNVFGRVPYTNTFLDQQAQERSMDKCLGWFLEPVVRGDYPFSMRVSA 355

DHRlike3_Sobic.008G080100 KYHKGADGRIGLALNVFGRVPYTNTFLDQQAQEMSMDKCLGWFLEPVLRGDYPFSMRVSA 357

*.*: * *** .****:**:* .** ***:****:*****.*** *.

DHR2_Sobic.008G080400 RDRLPHFTEKEQQKLVGSYDMIGINYYSSRFAKHVDITENFSPELNTHDCCATEEITGPN 414

DHRlike4_Sobic.008G080600 KDRVPYFKEIEQEKLVGSYDMIGIKLLH-------------------------------- 369

DHR1_Sobic.008G079800 RDRVPYFKEKEQEKLVGSYDMIGINYYTSTFSKHIDLSPNNSPVLNTDDAYASQETKGPD 415

DHRlike3_Sobic.008G080100 RDRLPYFKEKEQEKLVGSYDMIGINYYTSTFSKHNDISANYSPVLNTDDAYASQKTQGPD 417

:**:*:*.* **:***********:

DHR2_Sobic.008G080400 GNTIGPATGNAWVYMYPKGLKDILMIMKKRYGNPPVYITENGMGDIDNGDLSMEAALDDH 474

DHRlike4_Sobic.008G080600 -----LNTGNAWINMYPKGLHDILMTMKNKYGNPPMYITENGIGDIDKGDLPKALALEDH 424

DHR1_Sobic.008G079800 GNAIGPPTGNAWINMYPKGLHDILMTMKNKYGNPPMYITENGMGDIDKGDLPKPVALEDH 475

DHRlike3_Sobic.008G080100 GNAIGPPTGNAWINMYPKGLHDILMTMKNKYGNPPIYITENGIGDIDKGDLPKAVALEDH 477

*****: ******:**** **::*****:******:****:*** **:**

DHR2_Sobic.008G080400 IRLDYLQRHISVLKDSIDSGANVRGHFTWSLLDNFEWSSGYTERFGIVYVDRENGCKRTL 534

DHRlike4_Sobic.008G080600 TRLDYIQRHLSVLKQSIDLGANVRGYFAWSLLDNFEWSSGYTERFGIVYVDRDNGCERTM 484

DHR1_Sobic.008G079800 TRLDYIQRHLSVLKQSIDLGADVRGYFAWSLLDNFEWSSGYTERFGIVYVDRENGCERTM 535

DHRlike3_Sobic.008G080100 TRLDYIQRHLSVLKQSIDLGADVRGYFAWSLLDNFEWSSGYTERYGIVYLDRENGCERTM 537

****:***:****:*** **:***:*:****************:****:**:***:**:

DHR2_Sobic.008G080400 KRSARWLKEFNGAAKRPGNLIKPN-FSEINKIKVVTPA 571

DHRlike4_Sobic.008G080600 KRSAWWLQEFNGAAMKR--------------------- 501

DHR1_Sobic.008G079800 KRSARWLQEFNGAAKKVENNKILTPAGQLN-------- 565

DHRlike3_Sobic.008G080100 KRSARWFQEFNGAAKKKTQSKKA--------------- 560

**** *::****** :

**Fig. S2** Phylogenetic analysis of genes containing the cytochrome P450 domain PF0067 in *Sorghum bicolor*.

**
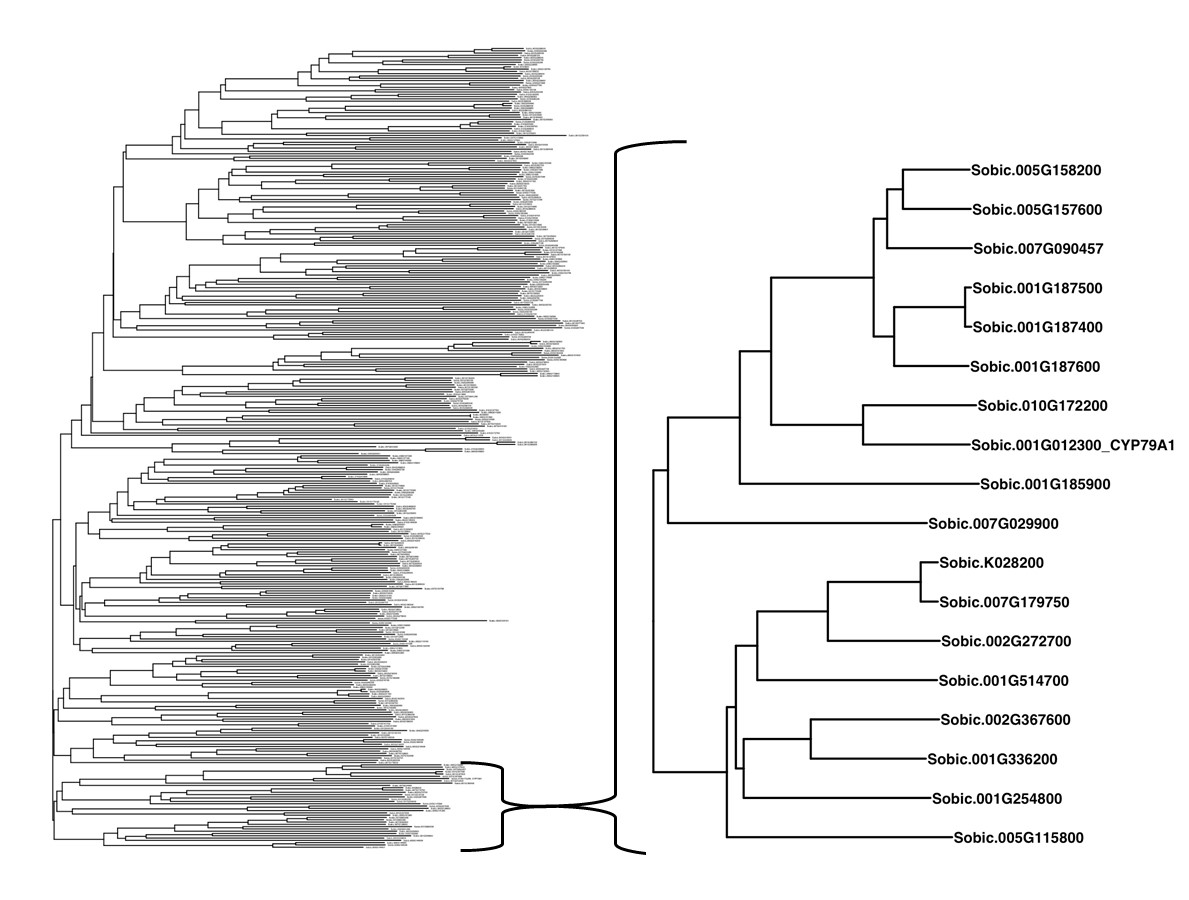
**

**Fig. S3** Phylogenetic analysis of genes containing the Glycoside hydrolase 1 domain (pf00232) in *Sorghum bicolor*.

**
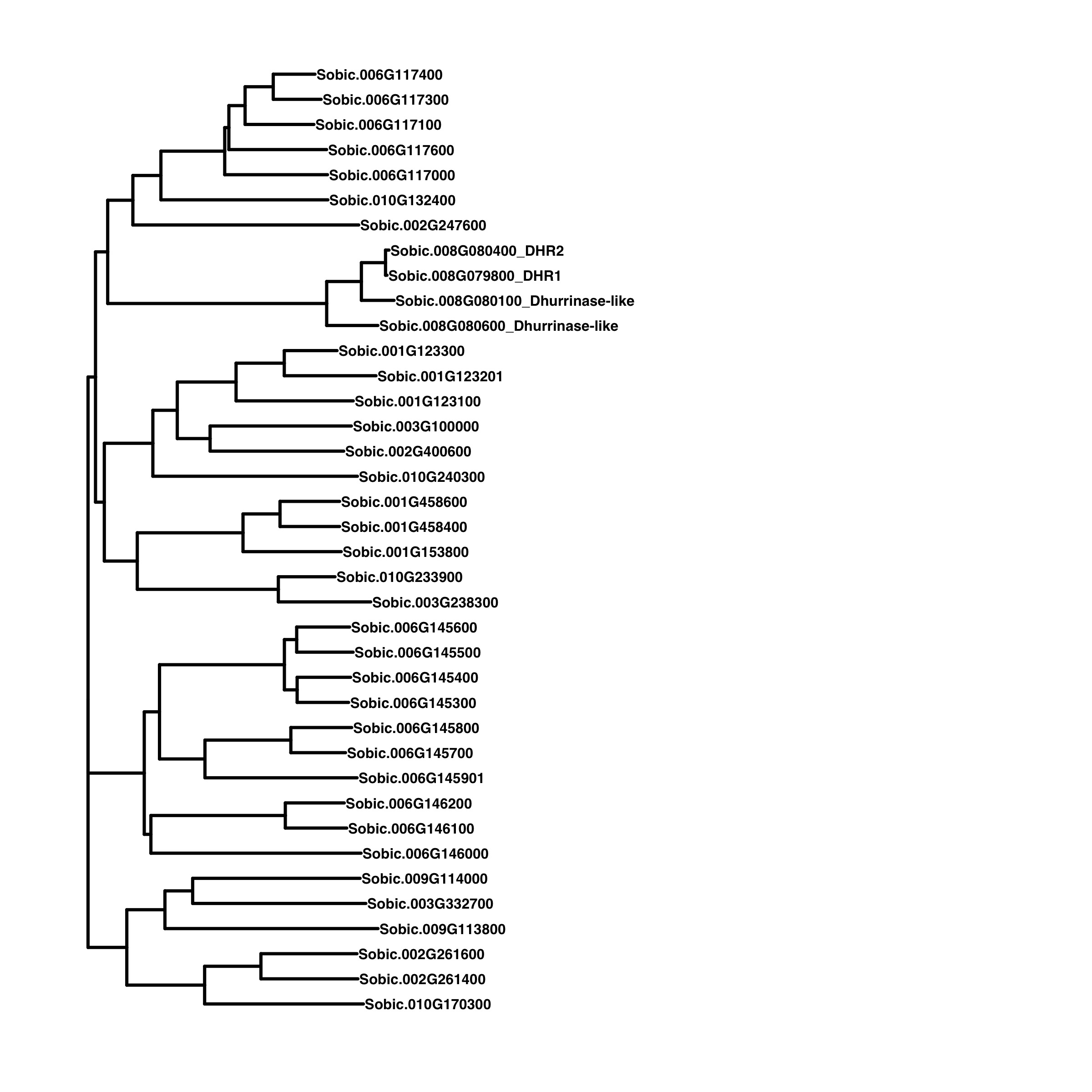
**

Dhurrinase and

Dhurrinase-like genes

**Fig. S4** Root sections collected for assay of HCNp. a)Root tip, 1.5cm; b) Elongation zone, 2.0cm; c) Mature zone 1, 4cm adjacent to section b; and d) Mature zone 2, 4cm adjacent to stem.


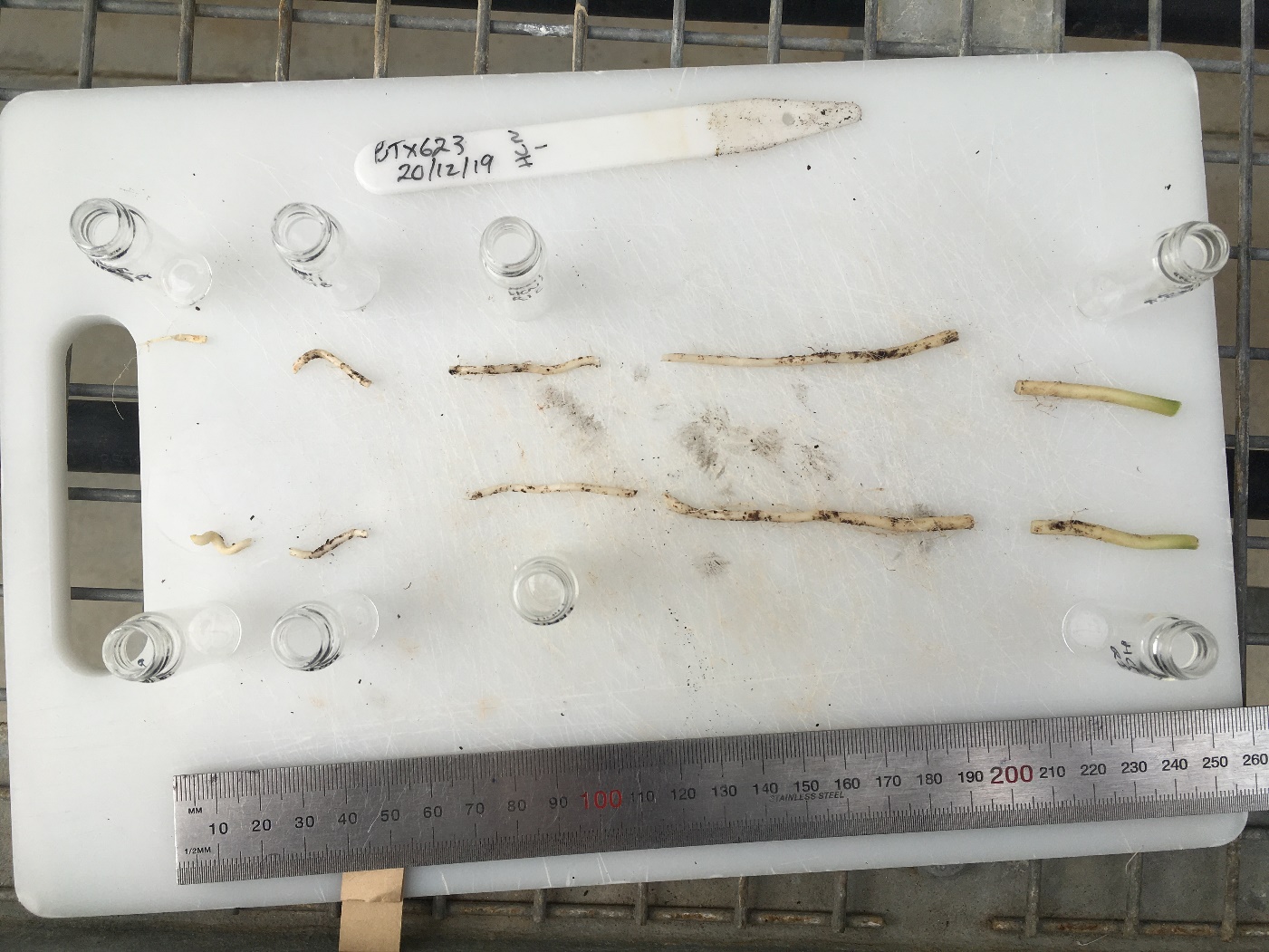


**aD**

**bD**

**cD**

1 cm

**dD**

**Tables S1-S7:** Included in Supplementary information as a separate excel file.

**Table S1** Heat map showing expression of transcripts for all cyanogenesis related genes in mature grain (dry seeds) and imbibed seed of BTx623, and tiller buds of the phytochrome B sorghum genotype 100M. TPM, transcripts per million reads, n = 3. Genes with increased expression levels are shown in blue and reduced expression levels in green.

**Table S2a** Heat map showing the expression of all cyanogenesis related genes in three different specific zones of cell development in nodal roots of S. bicolor TX08001. Gene expression was also analysed in the core and rind tissue of dormant roots of TX08001. Expression is given in Transcripts Per Million (TPM), n = 3. Regions with highest expression are shown in blue and lowest expression in green.

**Table S2b** Heat map showing the expression of all cyanogenesis related genes in root tissue of S. bicolor. Root tip of S. bicolor BTx623 sampled at four different stages of plant development. In addition the upper root section of juvenile (8 DAE) plants was also analysed. Expression is given in Transcripts Per Million (TPM), n = 3. Genes with increased expression levels are shown in blue and reduced expression levels in green.

**Table S3a** Heat map showing the expression of all cyanogenesis related genes in the first four internodes from the shoot apex of S. bicolor R.07020 at 60 DAE. Internodes were numbered from the sub-apical internodes 1-4 below the apical dome. Expression is given in Transcripts Per Million (TPM), n = 3.

**Table S3b** Heat map showing the expression of all cyanogenesis related genes in true stem tissue (i.e. outer leaf sheaths removed) of S. bicolor BTx623 at different stages of plant development: 2 mm juvenile stem at 8 DAE entire stem; 1 cm vegetative stem at 24 DAE entire stem; Floral initiation (44 DAE), 2 cm long growing internode near apex; mature internode from middle of the stem; Internode, mid stem anthesis (65 DAE); Internode, mid stem grain maturity (96 DAE). Expression is given in Transcripts Per Million (TPM), n=3.

**Table S4** Heat map showing the expression of all cyanogenesis related genes in leaf sheath tissue of two S. bicolor cultivars, TX08001 and BTx623, at different stages of plant development. Leaf sheath is defined as the outer leaf blades surrounding the internode stem sections. Expression is given in Transcripts Per Million (TPM), n = 3.

**Table S5** Heat map showing expression of all cyanogenesis related genes in leaves during S. bicolor BTx623 development. Juvenile, upper portion (distal) of third emerging leaf blade 8 DAE; Vegetative, middle portion of shoot after removing first through fourth emerged leaves 24 DAE; Floral initiation, upper portion (proximal) of leaf blade of last fully emerged leaf (ligulated) 44 DAE; Anthesis, upper portion (distal) of leaf blade of leaf below flag leaf 65 DAE; Grain maturity, upper leaf 96 DAE. Expression is given in Transcripts Per Million (TPM), n = 3.

**Table S6:** Heat map showing diel expression of all cyanogenesis related genes in leaves of *S. bicolor* BTx623 over a 36-hour period. Expression is given in Transcripts Per Million (TPM). Included in Supplementary information as a separate excel file.

**Table S7:** Phytozome and GenBank Gene IDs for the genes analyzed in this study.

**Methods S1** The selection of genes for transcript analysis based on identified roles in dhurrin metabolism. The following genes were selected for analysis of transcript levels based on identified functions in the dhurrin metabolism pathways (Fig. 1). The four genes involved in the biosynthesis of dhurrin, CYP79A1 (Sobic.001G012300.1), CYP71E1 (Sobic.001G012200.1), UGT85B1 (Sobic.001G012400.1), and the essential NADPH-dependent cytochrome P450 oxidoreductase, POR (Sobic.002G295100.1) (refs in ((Gleadow & Møller, 2014). The four identified dhurrinase genes, DHR1 (Sobic.008G079800.1), DHR2 (Sobic.008G080400.1), DHR-like3 (Sobic.008G080100.1) and DHR-like4 (Sobic.008G080600.1) and hydroxynitrile lyase (HNL, Sobic.004G335500.1) have been shown to be involved in the degradation of dhurrin (Hösel et al., 1987; Cicek & Esen, 1998; Krothapalli et al., 2013; Hayes et al., 2015; Nielsen et al., 2016). β-cyanoalanine synthase (CAS C1, Sobic.006G016900.1) and CAS 26 (Sobic.003G333700.1) were selected from the detoxification pathway (Nielsen et al., 2016; Akbudak et al., 2019). The nitrilases, NIT4A (Sobic.004G225200.1 ) and NIT4B2 (Sobic.004G225100.2) form a heterodimer and have been shown to be specifically involved in the turnover of dhurrin, whilst NIT4A and NIT4B1 (Sobic.004G225000.1) can also form heterodimers they are involved in the detoxification pathway (Jenrich et al., 2007). We also included genes postulated to be involved in dhurrin transport within or between cells; SbMATE2 (Sobic.001G012600.2 ) multidrug and toxic compound extrusion transporter (Darbani et al., 2016) and a putative member of the nitrate and peptide family (NPF) of transporters, SbCGTR1 (Sobic.001G133900.1). Jørgensen et al. (2017) identified MeCGTR1 in cassava and we used this gene and the PFAM:PF00854 and PTHR11654 specific domains relating to NPF transporters to search the sorghum genome and identified a potential candidate, SbCGTR1 (Sorghum bicolor cyanogenic glucoside transporter 1). Recently glutathione-S-transferases (GSTs) have been implicated in the endogenous dhurrin turnover pathway (Bjarnholt et al., 2018). Specifically two members of the lambda class of GSTs (GSTL1 Sobic.002G421200.2 and GSTL2 Sobic.009G033200.4) have been shown to be involved in the formation of p-hydroxyphenylacetonitrile, which is then converted to p-hydroxyphenylacetic acid and free ammonia by the NIT4A/NIT4B2 heterodimer. It is also possible that additional GSTs are involved in an earlier step in the endogenous turnover pathway so the expression of a GST (Sobic.001G012500) that co-clusters with the biosynthetic genes on chromosome 1 and two additional GSTs (GST1B Sobic.001G065800.1 and GST3 Sobic.003G416300.1) were also included in the study (Darbani et al., 2016; Nielsen et al., 2016).

**References**

**Akbudak MA, Filiz E, Uylas S. 2019.** Identification of O-acetylserine(thiol)lyase (OASTL) genes in sorghum (Sorghum bicolor) and gene expression analysis under cadmium stress. *Molecular Biology Reports* **46**(1): 343-354.

**Bjarnholt N, Neilson EHJ, Crocoll C, Jørgensen K, Motawia MS, Olsen CE, Dixon DP, Edwards R, Møller BL. 2018.** Glutathione transferases catalyze recycling of auto-toxic cyanogenic glucosides in sorghum. *The Plant Journal* **94**(6): 1109-1125.

**Cicek M, Esen A. 1998.** Structure and expression of a dhurrinase (beta-glucosidase) from Sorghum. *Plant Physiology (Rockville)* **116**(4): 1469-1478.

**Darbani B, Motawia MS, Olsen CE, Nour-Eldin HH, Møller BL, Rook F. 2016.** The biosynthetic gene cluster for the cyanogenic glucoside dhurrin in Sorghum bicolor contains its co-expressed vacuolar MATE transporter. *Scientific Reports* **6**(1): 37079.

**Gleadow RM, Møller BL. 2014.** Cyanogenic Glycosides: Synthesis, Physiology, and Phenotypic Plasticity. *Annual Review of Plant Biology* **65**(1): 155-185.

**Hayes CM, Burow GB, Brown PJ, Thurber C, Xin Z, Burke JJ. 2015.** Natural Variation in Synthesis and Catabolism Genes Influences Dhurrin Content in Sorghum. *The Plant Genome* **8**(2): plantgenome2014.2009.0048.

**Hösel W, Tober I, Eklund SH, Conn EE. 1987.** Characterisation of betaglucosidases with high specificity for the cyanogenic glucoside dhurrin in *Sorghum bicolor* (L.) Moench seedlings. *Arch. Biochem. Biophys.* **252**: 152-162.

**Jenrich R, Trompetter I, Bak S, Olsen CE, Møller BL, Piotrowski M. 2007.** Evolution of heteromeric nitrilase complexes in Poaceae with new functions in nitrile metabolism. *Proceedings of the National Academy of Sciences* **104**(47): 18848-18853.

**Jørgensen M, Xu D, Crocoll C, Ernst H, Ramírez D, Motawia M, Olsen C, Mirza O, Nour-Eldin H, Halkier B. 2017.** Origin and evolution of transporter substrate specificity within the NPF family. *eLife* **6**: e19466.

**Krothapalli K, Buescher EM, Li X, Brown E, Chapple C, Dilkes BP, Tuinstra MR. 2013.** Forward Genetics by Genome Sequencing Reveals That Rapid Cyanide Release Deters Insect Herbivory of Sorghum bicolor. *Genetics* **195**(2): 309-318.

**Nielsen LJ, Stuart P, Pičmanová M, Rasmussen S, Olsen CE, Harholt J, Møller BL, Bjarnholt N. 2016.** Dhurrin metabolism in the developing grain of Sorghum bicolor (L.) Moench investigated by metabolite profiling and novel clustering analyses of time-resolved transcriptomic data. *BMC Genomics* **17**: 1021.
